# The association between long working hours, shift work, and suicidal ideation: A systematic review and meta-analyses

**DOI:** 10.5271/sjweh.4182

**Published:** 2024-10-01

**Authors:** Joungsue Kim, Ria Kwon, Hyunok Yun, Ga-Young Lim, Kyung-Sook Woo, Inah Kim

**Affiliations:** 1 Department of Public Health Sciences, Hanyang University Graduate School, Seoul, Republic of Korea.; 2 Center for Cohort Studies, Total Healthcare Center, Kangbuk Samsung Hospital, Sungkyunkwan University School of Medicine, Seoul, Republic of Korea.; 3 Institute of Medical Research, School of Medicine, Sungkyunkwan University, Republic of Korea.; 4 Institute of Health and Society, Hanyang University, Seoul, Republic of Korea.; 5 Department of Occupational and Environmental Medicine, College of Medicine, Hanyang University, Seoul 04763, Republic of Korea.

**Keywords:** occupational risk factor, psychosocial risk factor, mental health, suicide, shift worker, nonstandard working hour

## Abstract

**Objectives:**

The association between occupational psychosocial factors and mental health has been studied in several systematic reviews. However, few systematic reviews exist that specifically address suicidal ideation. Therefore, this systematic review and meta-analysis aimed to examine the associations between long working hours, shift work, and suicidal ideation.

**Methods:**

We systematically screened and collected studies published between January 1970 and September 2022 from three electronic databases and Google Scholar. We conducted a meta-analysis using a random-effects model and assessed the risk of bias using a navigation guide. Additionally, the grading of recommendations assessment, development and evaluation (GRADE) approach was used to consider five items, including the risk of bias and publication bias.

**Results:**

Twenty-eight articles were included in this meta-analysis. After adjusting for covariates, the overall odds ratio (OR) for long working hours and shift work were 1.44 [95% confidence interval (CI) 1.26–1.63] and 1.34 (95% CI 1.22–1.47), respectively. Compared with those who worked <40 hours per week, those who worked >55 hours per week had a 1.65 (95% CI 1.37–1.98) higher risk of suicidal ideation, and fixed night shift workers had 1.37 (95% CI 1.03–1.83) higher risk than non-shift workers.

**Conclusions:**

Evidence has shown associations between long working hours, shift work, and suicidal ideation. Implementing evidence-based interventions to address these risk factors may help prevent the progression of suicidal ideation. However, further studies are critically needed, especially those using standardized exposure definitions and longitudinal data.

Suicidal ideation is a frequent and potentially life-threatening issue in public health, especially among individuals with severe mental disorders (1, 2). The burden of mental disorders peaks among middle-aged adults, the main working-age population (3), and suicide mortality has increased in this group (4). To prevent suicide in the working-age population, it is important to identify occupational risk factors for suicidal ideation as it is a strong predictor of attempting suicide within one to two years (5, 6).

While systematic reviews and meta-analyses have previously examined occupational factors and mental health (7–10), very few have specifically addressed suicidal ideation. Recent studies have provided evidence to support the associations between long working hours, shift work, and suicidal ideation among working-age individuals (11–15). Long working hours have a detrimental effect on the mental health of workers, leading to excessive fatigue and strain (16), and are linked to higher risks of sleep problems, risky alcohol consumption, depressive symptoms, and suicidal ideation (16–19). Similarly, shift workers are susceptible to insomnia, sleepiness, depression, and emotional dysregulation, all of which can increase their vulnerability to suicide (20–22). The mental health of workers is a crucial concern in the workplace because it can result in decreased productivity levels and higher rates of absenteeism (23). The factors that either exacerbate or mitigate the risk of developing psychosocial problems, such as depressive symptoms and suicidal ideation, may vary depending on the definition of long working hours, shift type, and sociodemographic characteristics (24). Therefore, it is important to take these factors into account when assessing the risk of suicidal ideation.

Long working hours and shift work have recently attracted attention as determinants of worker health in many countries, and their high prevalence continues to raise concerns regarding their potential impact on the health of large populations (25–27). Furthermore, the regulations for long working hours vary between countries, and there is no universally agreed-upon definition for the specific working conditions that qualify as “shift work”. The International Labour Organization (ILO) and the European Commission define long working hours as >8 hours/day or >48 hours/week (28, 29). It is notable that shift work is distinctly different from working “overtime” or “extended hours” (30, 31). The term “shift work” refers to a significant portion of working hours that is outside the standard Monday to Friday 08:00–09:00 to 16:00–17:00 hour schedule (30, 32).

Given the substantial amount of research conducted in recent years on the relationships between long working hours, shift work, and suicidal ideation, we aimed to conduct a systematic review to examine the comprehensive literature on this topic. We focused on obtaining results for each specific type of work, as different countries have different definitions of long working hours and different types of shift work, depending on the occupation. In addition, this systematic review aimed to assess the quality of the studies and, if possible, to summarize the findings through a meta-analysis.

## Methods

A meta-analysis was conducted to synthesize the results of individual quantitative studies examining the effects of long hours or shift work on suicidal ideation. The research process and the main content of the review were carried out in compliance with the Preferred Reporting Items for Systematic Reviews and Meta-Analyses (PRISMA) 2020 and the Meta-analyses of Observational Studies in Epidemiology (MOOSE) checklists (33, 34). The studies’ risk of bias was appraised using the Navigation Guide method (35) (supplementary material, www.sjweh.fi/article/4182, Appendix C. C1), and the overall evidence was determined using GRADE (supplementary Appendix D. S 4, 5).

### Search strategy and selection criteria

Using a strategy agreed upon by the researchers, we searched the electronic databases of Medline via PubMed, Embase, and CINAHL. To minimize bias in the search results, we also used Google Scholar, a well-known gray literature resource. The search terms for risk factors were “long working hours” and “shift work”, and the search term for outcome was “suicidal ideation”. We checked the MeSH terms and Emtree in each database and used Boolean operators and truncation to increase sensitivity and specificity. The filter function of the database was used for the search period and other restrictions (supplementary Appendix B. S 1, 2, 3). To identify articles not found in the database search, we reviewed the selected studies’ references and added the relevant article.

Four researchers independently selected the studies based on the inclusion and exclusion criteria, and inconsistencies were resolved through discussion. The initial selection or exclusion was based on the title, and the second on the abstract and full text; the reason for exclusion was recorded.

The inclusion criteria were: (i) studies in which the sample consisted of workers aged ≥18 years; (ii) studies that included long working hours and shift work as work-related factors; (iii) studies where suicidal ideation was measured by prevalence and an odds ratio based on “yes/no” answers, enabling quantitative synthesis; (iv) non-randomized studies (observational, cross-sectional, case-control, cohort, longitudinal, and before-and-after comparison); and (v) publication between 1 January 1970 and 30 September 2022. Owing to the high suicide rate in Korea, working hour regulations, and other factors, we limited the language to Korean in addition to English, aiming to include more Korean-specific literature.

Working >40 hours/week was considered to be long working hours and was compared to risk factors for standard working hours (35–40 hours), including side jobs (10). Studies that did not report working hours as a criterion for participation were identified through the results table, and those that included workers who worked <30 hours per week were excluded from the analysis. Shift work was taken to mean any work outside the standard weekly working hours (08:00–18:00 hours) (36).

### Data extraction and coding

To improve the accuracy of data extraction and coding, two reviewers extracted several articles and reviewed them with two other reviewers. Data were then extracted from the entire literature independently and cross-checked. When coding errors were found, the articles were scrutinized to resolve discrepancies until a consensus was reached.

The extracted information was divided into characteristics of the selected studies, characteristics for distinguishing exposure risks, and information for calculating the effect size. General study characteristics included authors, publication year, country, data source, and sample size. Participants’ characteristics included sex, age, and occupation. The information used to calculate the integrated effect value included the effect values [odds ratios (OR), risk ratio (RR)] for work hours, shift work type, and suicidal ideation, as well as statistical information [95% confidence interval (CI), significance level, and covariate]. Estimates of sex and geography/work type were extracted to analyze the subgroups.

### Meta-analytical methods

Studies report different forms of estimating risk (37); therefore, a common measure is needed to aggregate the results. To estimate the pooled risk using the most commonly reported OR in the selected literature, studies that did not report OR but could be converted were converted to OR. We analyzed the unadjusted and adjusted models separately and interpreted significant results focusing on the most tightly adjusted model, including socio-demographic and occupational factors. The effect value and 95% CI of each study were log-transformed to obtain the integrated value, which was then converted back to OR for interpretation. We calculated inverse variance weights using standard errors reported in the studies (35). As this review included primary studies from different countries, industries, and types of work, a random-effects model was used to control for variability among the included studies (38, 39).

We created subgroups using categories such as sex and geographic region (Asian and non-Asian) (40). For the analysis of long working hours, we divided working time into four groups: <40 (ref group), 41–48, 49–54, and ≥55 hours (10). According to the ILO, about 36.1% of workers worldwide work ≥48 hours per week (41); therefore, we also compared the risk of suicidal ideation between those working <48 hours and those working >48 hours (maximum weekly hours).

For shift work analysis, we defined shift work outside of a regular daytime schedule and included irregular schedules, split day shifts, evening shifts, night shifts, rotating shifts, and other schedules (42). Most studies included in the analysis were either binary (yes/no) or compared day shifts with other shifts. However, three studies distinguished between fixed evening and fixed night shifts; therefore, we conducted a subgroup analysis to determine whether there were differences based on the type of shift. While some studies have reported that fixed night shifts may have fewer health effects due to the adjustment of biological circadian rhythms (43), others have shown that of all shift types, workers on fixed night shifts have the shortest sleep duration and the highest prevalence of insomnia and mental health problems (44). Therefore, the fixed night shift group was analyzed separately in this study. Shift work is common in a variety of industries, including public safety, manufacturing, and healthcare (9, 45). We focused on manufacturing because of the prevalence of shift work in the industry and the high rates of short sleep duration among manufacturing workers (46). Thus, we conducted a subgroup analysis by classifying manufacturing and non-manufacturing industries.

Funnel plots were used for the assessment of publication bias, and asymmetry in the funnel plots was checked through Egger’s test (47). If publication bias was present, we adjusted for missing studies with the trim-and-fill process to transform asymmetry into symmetry and check whether the overall effect size and CI improved. In addition, a sensitivity analysis was performed to ensure that the results of the analysis were consistent across different subgroups. A R version 4.1.2 “meta” package was used for the statistical analysis.

## Results

**Figure 1:**
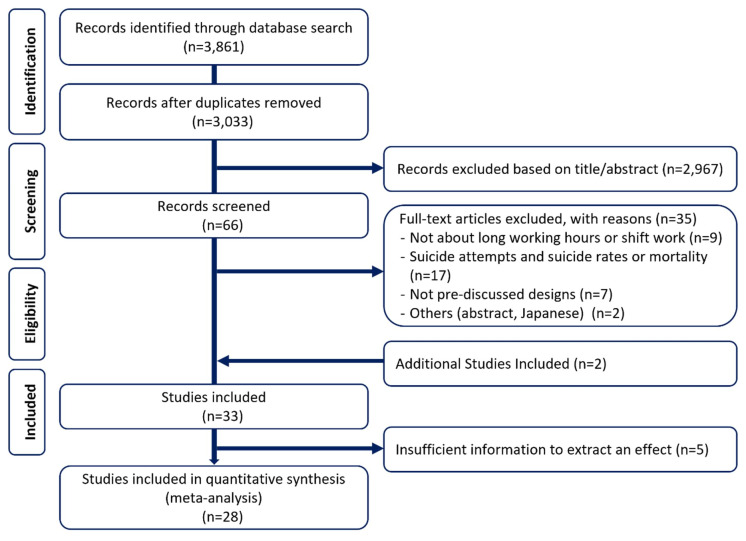
PRISMA flow chart for inclusion in the meta-analysis.

A total of 31 articles were selected in accordance with the inclusion and exclusion criteria of this study, and 2 were added by reviewing the references of the selected studies (16, 48). It was observed that 4 studies could not be standardized to comparable effect sizes (16, 48–50) and 1 used different criteria for working hours (51) (figure 1). Therefore, 5 studies were excluded from the meta-analysis.

### Characteristics of included studies

The characteristics of the 33 studies identified for this review are as follows (table 1a–b): 26 studies on suicidal ideation among long working hours and shift workers were conducted in Asia (23 of these in three countries), while only 7 were conducted in non-Asian countries. Most studies used single data from the country in which the study was done, undertaking cross-sectional research. Most studies included both sexes, with three studies involving only women, and one study involving only men. The sample sizes of the 33 studies in the review varied from 111 to 79 009 participants. Unless a specific occupational group was investigated, the industry or occupation was not reported, and the majority of employment types were full-time workers. Of the 16 studies analyzing the effect of long working hours on suicidal ideation, 9 showed the effect of shift work, and 8 showed the effect of both. To ascertain the association between long working hours or shift work and suicidal ideation, 17 studies considered mental health factors such as depression, and 29 studies considered occupational factors.

**Table 1a:** Characteristics of included studies. [NR=not reported; LWH=long working hours; SW=shift work.]

Author, year (ref)	Country	Geographic location	Study design	Data source	Data collection	Gender, mean age	Sample size
**Exposure: LWH**							
Tyssen et al 2001 (90)	Norway	National	Prospective cohort	Cohort	1993-1994 (mean f/u 1 year)	Both, 28	522
Al-Maskari et al 2011 (51)*	United Arab Emirates	Local	Cross sectional	Survey	2008	Men, NR	298
Langhinrichsen-Rohling et al 2011 (91)^†^	USA	National	Cross sectional	Community Assessment survey	2006	Both, 31.8	52 780
Kim et al 2012 (48)*	Korea	National	Cross sectional	The Korean National Health and Nutrition Examination Surveys	2007-2009	Both, 41.4	4539
Choi 2018 (13)	USA	National	Cross sectional	Midlife Development in the United States study	2004-2006	Both, NR	582
Liu et al 2019 (49)*	China	Local	Cross sectional	Survey	2017	Both, NR	2063
Park et al 2020 (58)	Korea	National	Cross sectional	The Youth Panel	2012	Both, NR	3332
Petrie et al 2020 (14)	Australia	National	Cross sectional	Beyond Blue National Mental Health Survey	2013	Both, NR	2706
Choi et al 2021 (19)	Korea	National	Cross sectional	The Korean National Health and Nutrition Examination Surveys	2014, 2016, 2018	Both, NR	7082
Kim et al 2021 (92)	Korea	National	Cross sectional	The Korean National Health and Nutrition Examination Surveys	2007-2018	Both, NR	13 628
Malone et al 2021 (93)	USA	National	prospective cohort	The Intern Health Study	2012-2014, 2015	Both, 27.4	4336
Xu et al 2021 (50)*	China	Local	Cross sectional	Survey	2020	Both, 33.4	11 507
Ishikawa 2022A (64)	Japan	National	Cross sectional	Survey	2020	Both, NR	4306
Ishikawa 2022B (94)	Japan	National	Cross sectional	Survey	2019	Both, NR	919
Lee et al 2022 (16)*	Korea	Local	Cross sectional	Survey	2015-2019	Both, 39.5	9326
Li et al 2022 (95)	China	Local	Cross sectional	The Routine Health Check-up cohort	NR	Both, 37.0	4136
**Exposure: SW**							
Violanti et al 2008 (96)^†^	USA	Local	Cross sectional	Survey	1994-2000	Both, NR	111
Takusari et al 2011 (15)	Japan	Local	Cross sectional	Survey	2008	Both, NR	3233
Kang et al 2017 (97)	Korea	Local	Cross sectional	Survey	2015	Both, NR	14 104
Kim et al 2019 (11)	Korea	National	Cross sectional	The Korean National Health and Nutrition Examination Surveys	2008-2016	Both, NR	17 756
Park 2019 (98)	Korea	National	Cross sectional	The Korean National Health and Nutrition Examination Surveys	2015	Both, 44.7	2364
Ahn et al 2020 (99)^†^	Korea	Local	Cross sectional	Survey	2017	Both, NR	836
Son & Lee 2021 (100)	Korea	Local	Cross sectional	Survey	2014	Women, NR	508
Kim et al 2022 (101)	Korea	National	Cross sectional	The Korean National Health and Nutrition Examination Surveys	2007-2018	Both, NR	33 047
Park et al 2022 (24)	Korea	Local	Cross sectional	The Kangbuk Samsung Cohort	2014-2015	Both, NR	79,009
**Exposure: LWH+SW**							
Yoon A et al 2015 (12)	Korea	National	Cross sectional	The Korean National Health and Nutrition Examination Surveys	2007-2012	Both, NR	12 076
Yoon B et al 2015 (102)	Korea	National	Cross sectional	Korea Community Health Survey	2008	Both, NR	67 471
Chin et al 2018 (103)^†^	Taiwan	National	Cross sectional	Survey	NR	Women, 32.7	2734
Kim et al 2020 (104)^†^	Korea	Local	Prospective cohort	The Kangbuk Samsung Cohort	2012-2017 (mean f/u 3.82 year)	Both, NR	75 786
Lin et al 2020 (105)	China	Local	Cross sectional	Survey	2015	Women, 31.6	968
Niedhammer et al 2020 (106)	France	National	Cross sectional	French National Working Conditions Survey	2016	Both, NR	20 430
Bryant-Lees et al 2021 (107)^†^	USA	National	Cross sectional	Survey	2018	Both, NR	789
Han et al 2021 (108)	Korea	National	Cross sectional	The Korea National Health and Nutrition Examination Surveys VI and VII	2013-2018	Both, NR	14 625

* Exclude from meta-analysis. ^†^ Report proportions only (unadjusted effects of integrations).

**Table 1b:** Exposure and outcome assessment of included studies. [NR=not reported; h/w= hours per week; h/d=hours per day; LWH=long working hours; SW=shift work; SC=sociodemographic characteristics; HB=health behaviors; PH=physical health; MH=mental health; OF=occupational factors]

Author, year (ref)	Occupation or job status	Exposure	Standard working conditions	Comparison of working conditions	Period for outcome index	Adjusted confounding
Tyssen et al 2001 (90)	Physicians	LW	Weekly hours worked (continuous)	NR	Suicidal ideation in the past year	SC, HB, MH, OF
Al-Maskari et al 2011 (51)*	Immigrant workers (construction, garage mechanic, carpenter, other)	LWH	<8 h/d	≥8 h/d	Ever thought about suicide	SC, HB, OF
Langhinrichsen-Rohling et al 2011 (91)^†^	Armed forces (pilot, logistics, support, other operations, medical, acquisition, professional, other)	LWH	Weekly hours worked (continuous)	NR	Suicidal ideation in the past year	SC, MH, OF
Kim et al 2012 (48)*	Full-time worker	LWH	40–51 h/w	<40, 52–59, ≥60 h/w	Suicidal ideation in the past year	SC, MH, OF
Choi 2018 (13)	Full-time or part-time workers	LWH	≤40 h/w	≥41 41–48, 49-56, ≥57 h/w	Suicidal ideation in the past year	SC, OF
Liu et al 2019 (49)*	Medical staff /non-medical staff	LWH	Weekly hours worked (continuous)	NR	Suicidal ideation in the past year	SC, MH, OF
Park et al 2020 (58)	Full-time worker	LWH	31–40 h/w	41–50, 51-60, >60 h/w	Suicidal ideation in the past year	SC
Petrie et al 2020 (14)	Full-time worker	LWH	40–44 h/w	35–39, 45–49, 50-54, >55 h/w	Suicidal ideation in the past year	SC, OF
Choi et al 2021 (19)	Work >35 h/w	LWH	40 h/w	35–39, 41–52, 53–68, ≥69 h/w	Ever thought about suicide	SC, HB, PH, OF
Kim et al 2021 (92)	Wage workers, owner-operators & employers, unpaid family worker	LWH	≤40 h/w weekly hours worked (continuous)	40.1–52, >52 h/w NR	Suicidal ideation in the past year	SC, HB, MH, OF
Malone et al 2021 (93)	Physicians (medical interns)	LWH	Weekly hours worked (continuous)	NR	Suicidal ideation in the past 2 weeks	SC, HB, MH, OF
Xu et al 2021 (50)*	Hospital staff (doctor, nurse, technician, administrator)	LWH	Weekly hours worked (continuous)	NR	Suicidal ideation in the past 2 weeks	SC, MH, OF
Ishikawa 2022A (64)	Physicians	LWH	<40 h/w	40–59, 60–79, 80–99, ≥100 h/w	Suicidal ideation in the past week	SC, OF
Ishikawa 2022B (94)	Physicians (obstetrician/gynaecologists)	LWH	<59 h/w	60–79, 80–99, ≥100 h/w	Suicidal ideation in the past week	SC, OF
Lee et al 2022 (16)*	Work >40 h/w	LWH	Weekly hours worked (continuous)	NR	Suicidal ideation in the past year	SC, MH, OF
Li et al 2022 (95)	NR	LWH	(Overtime) rarely or never	(overtime) occasionally, frequently	Suicidal ideation in the past year	SC, HB, PH, MH, OF
Violanti et al 2008 (96)^†^	Police officers	SW	% hours on SW: Day: 0.19–63.16 Afternoon: 0–1.37 Midnight: 0–0.76 Total h/w: 18.55–28.38	% hours on SW: Day: 64.58–91.57, 92.39–99.74 Afternoon: 1.39–13.35, 15.64–98.85 Midnight: 0.96–6.05, 6.94–97.86 Total h/w: 28.98–34.39, 34.49–38.09	Ever thought about suicide	SC, OF
Takusari et al 2011 (15)	Workers from medium-sized business	SW	Non-SW	SW	Ever thought about suicide	SC, HB, MH, OF
Kang et al 2017 (97)	Workers of an electronic manufacturer	SW	Daytime work	SW	Suicidal ideation in the past year	SC, OF
Kim et al 2019 (11)	NR	SW	Daytime work	evening, night shift	Suicidal ideation in the past year	SC, PH, MH, OF
Park 2019 (98)	Waged employees	SW	Non-SW	SW	Suicidal ideation in the past year	SC, HB, PH, MH, OF
Ahn et al 2020 (99)^†^	Display manufacturing workers	SW	Non-SW	SW	Suicidal ideation in the past year	SC, HB, MH, OF
Son & Lee 2021 (100)	Worker at an electronics company	SW	Non-SW	yes, shift: 4 × 8 h or 3 × 12 h	Suicidal ideation in the past year	SC, HB
Kim et al 2022 (101)	Wage workers, owner-operators & employers, unpaid family worker	SW	Daytime work	shift work(total), fixed evening, fixed night, regular day and night rotating, 24-h rotating, split, irregular rotating	Suicidal ideation in the past year	SC, HB, MH, OF
Park et al 2022 (24)	NR	SW	Non-SW	shift work	Suicidal ideation in the past year	SC, MH, OF
Yoon A et al 2015 (12)	Work >35 h/w	LWH+SW	LW: <52 h/w, SW: daytime work	LW: 52–59, ≥60 h/w SW: shift or night work	Suicidal ideation in the past year	SC, HB, PH, OF
Yoon B et al 2015 (102)	NR	LWH+SW	LW: 35-40 h/w, SW: daytime work	LW: 41-50, 51-60, > 60 h/w SW: shift or night work	Suicidal ideation in the past year	SC, HB, PH, OF
Chin et al 2018 (103)^†^	Full- time nurses	LWH+SW	LW: 40–45 h/w SW: day & evening shift in 3 months	LW: 46-60, > 60 h/w SW: night shift and rotating shift	Ever thought about suicide	SC, HB, MH
Kim et al 2020 (104)^†^	NR	LWH+SW	LW: ≤40 h/w, SW: non-shift work	LW: 41–50, ≥ 51 h/w SW: shift work	Suicidal ideation in the past year	SC, HB, PH
Lin et al 2020 (105)	Physicians, nurses, school teachers, bank employees	LWH+SW	LW: ≤40 h/w, Night SW: no	LW: > 40 h/w Night SW: yes	Suicidal ideation in the past 2 weeks	SC, HB, OF
Niedhammer et al 2020 (106)	Working population	LWH+SW	LW: ≤48 h/w, SW: non-shift work	LW: >48 h/w SW: yes	Suicidal ideation in the past year	SC, OF
Bryant-Lees et al 2021 (107) ^†^	US Air Force	LWH+SW	LW: <50 h/w SW: Standard day	LW: ≥50 h/w SW: yes	Ever thought about suicide	SC, HB, MH, OF
Han et al 2021 (108)	Work more than 30 h/w	LWH+SW	LW: 31–40 h/w, SW: non-shift work	LW: 41–50, 51–60, >60 h/w SW: yes	Suicidal ideation in the past year	SC, HB, PH, OF

* Exclude from meta-analysis. † Report proportions only (unadjusted effects of integrations).

### Risk of bias at the individual study level

Four authors independently analyzed the risk of bias for each study included in the analysis and organized these into two groups. In instances of disparities, consensus was achieved through discussion or consultation. We evaluated the risk of bias by examining information obtained from the records of the studies included in our analysis. The risk of bias rating for each domain, across all 33 included studies, is displayed in table 2. The justification for each assessment of every domain, can be found in the risk of bias table in supplementary Appendix C.

**Table 2a:** Study/Navigation Guide risk of bias ratings. [PL=probability low; PH=probability high.]

Exposure	Long working hours															
Navigation Guide (Woodruff & Sutton 2014). Risk of bias domain	Tyssen (2001)	Al-Maskari (2011)	Langhin-richsen-Rohling (2011)	Kim (2012)	Choi (2018)	Liu (2019)	Park (2020)	Petrie (2020)	Choi (2021)	Kim (2021)	Malone (2021)	Xu (2021)	Ishikawa		Lee (2022)	Li (2022)
													(2022A)	(2022B)		
1. Are the study groups at risk of not representing their source populations in a manner that might introduce selection bias?	PL	PH	Low	Low	Low	PL	Low	PL	Low	Low	PL	PL	PL	PL	Low	Low
2. Was knowledge of the exposure adequately prevented (i.e. blinded or masked) prevented during the study,potentially leading to subjective measurement of either exposure or outcome?	PL	PH	Low	Low	PL	Low	Low	Low	Low	Low	PL	Low	PL	PL	Low	Low
3. Were exposure assessment methods lacking accuracy?	Low	Low	Low	Low	PL	PL	Low	PL	Low	Low	PH	PL	PH	PL	Low	PL
4. Were outcome assessment methods lacking accuracy?	Low	PL	Low	Low	Low	Low	Low	Low	Low	Low	Low	Low	Low	Low	Low	Low
5. Was potential confounding inadequately incorporated?	PL	Low	PL	Low	PL	PL	PL	Low	Low	Low	Low	Low	Low	Low	Low	PL
6. Were incomplete outcome data inadequately addressed?	PL	PL	Low	Low	Low	Low	Low	PL	Low	Low	Low	PL	PH	PL	Low	PL
7. Does the study appear to have selective outcome reporting?	Low	PL	Low	Low	Low	PL	Low	PL	Low	Low	Low	Low	PL	Low	Low	Low
8. Did the study receive any support from a company, study author, or other entity having a financial interest in any of the exposures studied?	Low	Low	Low	Low	Low	Low	Low	Low	Low	Low	Low	Low	Low	Low	Low	Low
9. Did the study appear to have other problems that could put it at a risk of bias?	PL	PL	Low	PL	PL	PL	PL	PL	Low	Low	PL	Low	PL	PL	PL	PL

**Table 2b:** Study/Navigation Guide risk of bias ratings.[PL=probability low; PH=probability high.]

Exposure	Long working hours																
Navigation Guide (Woodruff & Sutton 2014). Risk of bias domain	Violanti (2008)	Takusari (2011)	Kanget (2017)	Kim (2019)	Park (2019)	Ahn (2020)	Son & Lee (2021)	Kim (2022)	Park (2022)	Yoon		Chin (2018)	Kim (2020)	Lin (2020)	Nied-hammer (2020)	Bryant-Lees (2021)	Han (2021)
										A (2015)	B (2015)						
1. Are the study groups at risk of not representing their source populations in a manner that might introduce selection bias?	Low	PL	PL	Low	Low	PL	PL	Low	Low	Low	Low	PH	PL	PL	Low	PL	Low
2. Was knowledge of the exposure adequately prevented (i.e. blinded or masked) prevented during the study,potentially leading to subjective measurement of either exposure or outcome?	Low	Low	Low	Low	PL	Low	PL	Low	Low	Low	Low	Low	Low	PL	Low	Low	Low
3. Were exposure assessment methods lacking accuracy?	Low	Low	Low	Low	Low	PL	Low	PL	Low	Low	PL	PL	Low	Low	Low	Low	Low
4. Were outcome assessment methods lacking accuracy?	Low	Low	Low	Low	Low	Low	Low	Low	Low	Low	Low	Low	Low	Low	Low	Low	Low
5. Was potential confounding inadequately incorporated?	PL	Low	Low	Low	Low	Low	PL	Low	PL	Low	Low	PL	Low	Low	Low	PL	Low
6. Were incomplete outcome data inadequately addressed?	Low	PL	Low	Low	Low	Low	Low	Low	Low	Low	Low	Low	Low	Low	Low	PL	Low
7. Does the study appear to have selective outcome reporting?	Low	Low	Low	Low	Low	Low	Low	Low	Low	Low	Low	Low	Low	Low	Low	PL	Low
8. Did the study receive any support from a company, study author, or other entity having a financial interest in any of the exposures studied?	Low	Low	PL	Low	Low	Low	Low	Low	Low	Low	Low	Low	Low	Low	Low	PL	Low
9. Did the study appear to have other problems that could put it at a risk of bias?	PL	Low	Low	Low	PL	Low	PL	Low	PL	Low	Low	PL	Low	Low	Low	PH	Low

In all studies, workers self-reported suicidal ideation, which may lead to detection bias. However, we believe the risk of bias is low because almost all the studies utilized valid and reliable scales.

### Suicidal ideation linked to long working hours

A total of 24 studies estimates were reported on the effect of long working hours on the risk of suicidal ideation. In the pooled analysis, working longer hours significantly elevated the risk of suicidal ideation (OR 1.44, 95% CI 1.26–1.63) (figure 3). Comparable results were found for men-only, women-only, Asian, and non-Asian groups.

Regarding standard working hours, those who worked ≥55 hours per week were significantly more at risk of suicidal ideation compared to those who worked ≤40 hours per week (OR 1.65, 95% CI 1.37–1.98). However, no statistically significant risk was identified in the two groups working <55 hours (41–48 and 49–54 hours). Considering the maximum hours worked per week, those working >48 hours were significantly more exposed to suicidal ideation than those working ≤48 hours (OR 1.62, 95% CI 1.34–1.96).

**Figure 2:**
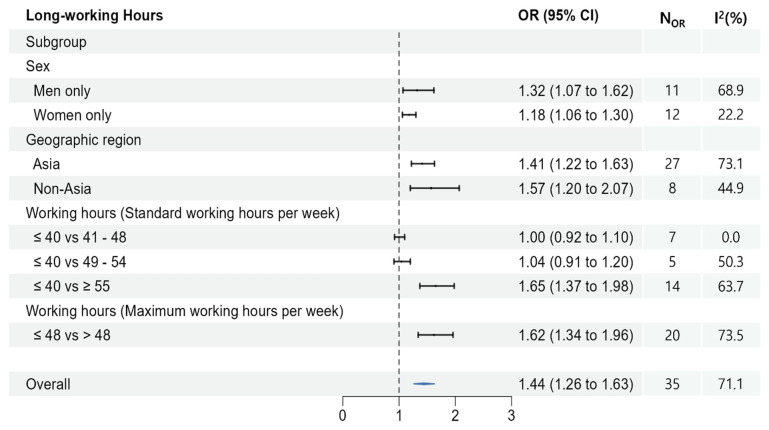
Meta-analyses of the association between long working hours and suicidal ideation.

**Figure 3:**
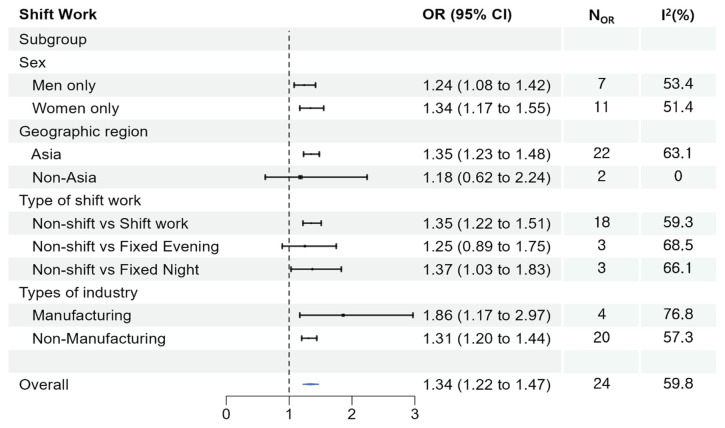
Meta-analyses of the association between shift work and suicidal ideation.

### Suicidal ideation linked to shift work

The pooled analysis showed that shift work significantly raised the risk of suicidal ideation (OR 1.34, 95% CI 1.22–1.47) (figure 3). Similar results were observed for men- and women-only groups. In the subgroups, shift workers and fixed night workers had a higher risk of suicidal ideation compared to those who did not work shifts (OR 1.35, 95% CI 1.22–1.51 and OR 1.37, 95% CI 1.03–1.83, respectively). In addition, both manufacturing and non-manufacturing workers were at a higher risk of suicide compared to those who did not work shifts (OR 1.86, 95% CI 1.17–2.97 and OR 1.31, 95% CI 1.20–1.44, respectively).

### Publication bias and sensitivity analysis

**Figure 4:**
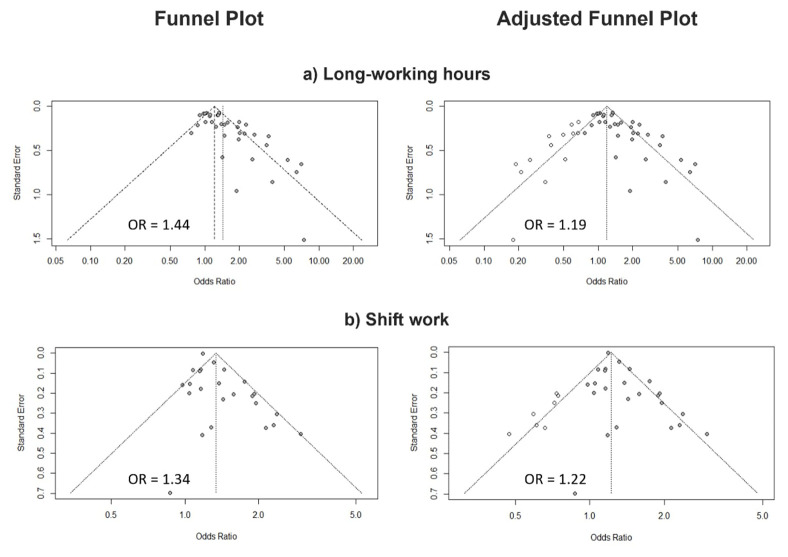
Publication bias of the effect of long working hours or shift work and suicidal ideation.

In the study on long working hours and suicidal ideation, the asymmetry of the funnel plot indicated a possible publication bias, which was confirmed by Egger’s test (P<0.0001). If there was a publication bias, the trim-and-fill method was used to estimate the adjusted values (52). After adding 13 studies to convert asymmetry to symmetry, the results showed that those who worked long hours had a 18% higher rate of suicidal ideation compared to those who did not (OR 1.18, 95% CI 1.00–1.41). This was <44% before adjustment (OR 1.44); but was still statistically significant. Similarly, publication bias was identified in the results on shift work and suicidal ideation using the Egger’s test (P=0.004). The adjusted results of adding the 7 studies showed that shift workers were 22% more likely to have suicidal ideation than non-shift workers (OR 1.22, 95% CI 1.08–1.38), which was lower than the unadjusted 34% (OR 1.34), but statistically significant (figure 4). Sensitivity analyses from main meta-analysis on the long working hours showed no significant differences by risk of bias (only “low”/ “probably low” versus any “high”/ “probably high”), study design (cross-sectional versus cohort), and adjustment for mental health factors (with versus without). However, there were meaningful differences by period for outcome index and study conducted country. The risk of suicidal ideation during the past week (or two weeks) was reported to be higher than during other periods, although this effect was not found to be significant. Furthermore, studies conducted outside of Korea demonstrated a greater increase in effect estimates than those conducted in Korea. Sensitivity analyses from the main meta-analysis on the shift work showed no significant differences by period for outcome index and by the study conducted country (table 3, supplementary Appendix G, figure G1~8).

**Table 3:** Summary of main results from sensitivity analysis on suicide ideation and long working hours, shift work.

Long-working hours					Shift work			
Risk of bias			P=0.07		Risk of bias			
	Only “low”/“probably low”	1.40	1.23–1.59			Only “low”/“probably low”	-	-
	Any “high”/“probably high”	3.03	1.33–6.94			Any “high”/“probably high”	-	-
Study design			P=0.23		Study design			
	Cross-sectional	1.40	1.25–1.57			Cross-sectional	-	-
	Cohort	1.12	0.79–1.58			Cohort	-	-
Period for outcome index			P=0.04		Period for outcome index			P=0.29
	Week	2.30	0.97–5.42			Week	1.95	1.20–3.18
	Year	1.28	1.14–1.44			Year	1.25	1.21–1.45
	Ever thought	1.96	1.37–2.80			Ever thought	1.10	0.530–2.64
Adjustment for mental health factors			P=0.14		Adjustment for mental health factors			P=0.87
	With	1.14	0.83–1.57			With	1.36	1.20–1.54
	Without	1.48	1.29–1.71			Without	1.34	1.15–1.56
Study conducted country			P=0.00		Study conducted Country			P=0.48
	Korea	1.23	1.10–1.37			Korea	1.33	1.21–1.46
	Other	1.89	1.50–2.39			Other	1.52	1.06–2.17

## Discussion

We completed a systematic review and meta-analysis of 28 articles to examine the associations between long working hours, shift work, and suicidal ideation. The results showed that employees exposed to long working hours faced a 1.44 times higher likelihood of experiencing suicidal ideation than those with standard working hours. Similarly, shift workers were 1.34 times more likely to report suicidal ideation compared to their daytime-working counterparts.

These findings support the existing literature and provide further evidence of the association between occupational factors, including long working hours and shift work, and an increased hazard of suicidal ideation.

In the existing literature, we found that in addition to long working hours, emotional demands, emotional exhaustion, and burnout were significantly associated with suicidal ideation (53, 54). However, it is important to note that working long hours, including shift work, increases an individual’s exposure to psychosocial or physical stressors that may arise at work. This, in turn, amplifies the detrimental effects of other negative working conditions (55). Therefore, we want to emphasize that working time arrangements should be recognized as a contributing factor to suicidal ideation.

### Long working hours

Very long working hours (≥55 hours/week) were associated with a higher risk of suicidal ideation (OR 1.65, 95% CI 1.37–1.98) compared with standard working hours (<40 hours/week). Furthermore, individuals working >48 hours faced an increased risk of suicidal ideation (OR 1.62, 95% CI 1.34–1.96) compared with those working <48 hours (the legal maximum hours in many countries). Long working hours are reportedly associated with mental health problems (56–58) and it is possible that this increases the incidence of suicidal ideation. Long working hours lead to weakened social support because of less time spent with family and friends (59) and recovery time from work not being guaranteed (60). However, a recent meta-analysis reported a lack of evidence that long working hours are detrimental to the risk of depression (10), indicating that more research is needed.

According to data from 2016, Southeast Asia had the highest percentage of people working ≥55 hours/week (11.7%). This was approximately three times higher than that in Europe (3.5%) (61). Moreover, this review includes only two studies conducted in European countries, which is likely to be a result of the implementation of the Working Time Directive within the European Union. This directive aims to enhance working practices pertaining to working hours and holidays (62). However, we observed that suicidal ideation was higher among individuals with long working hours in both Asian and non-Asian countries, despite variations in the definition of long working hours across regions. This implies that long working hours are an established risk factor for suicidal ideation, regardless of work culture, institution, and other factors.

Furthermore, a subgroup analysis revealed an elevated risk of long working hours among both men (OR 1.32, 95% CI 1.07–1.62) and women (OR 1.18, 95% CI 1.06–1.30). Previous findings in the field have demonstrated some inconsistencies (63–65). The dissimilarities in lifestyle, working environment, and employment type among individuals may help explain this divergence (15). Additionally, caution needs to be exercised when interpreting these results due to variations in work time arrangements based on sex in the workplace (66). Men also tend to have standard employment and work longer hours and, are more possible to experiencing mental health problems (67–69). Furthermore, women’s long working hours can be attributed to the potential double burden they face when combining their paid working hours with their household working hours (70).

### Shift work

An analysis of shiftwork types revealed that the group with fixed night shifts had the highest risk of suicidal ideation (OR 1.37, 95% CI 1.03–1.83). This aligns with previous findings indicating that continuous exposure to night shifts alone results in a greater risk, potentially due to the higher prevalence of insomnia and minor mental disorders (44). However, in some cases, no significant differences were found (71). Inconsistencies in the existing literature may be attributed to a lack of clarity in distinguishing between shift work types and a failure to account for potential confounding variables. Therefore, further research distinguishing between fixed night shifts, shifts that include night shifts, or other work schedules is needed to study the risk of night work for suicidal ideation. Furthermore, it is crucial to consider selection effects when investigating the causal relationships between mental health and night work. Individuals with existing mental health problems may be more likely be selected (or self-selected) into night work as a form of employment (72, 73). These selection effects have the potential to overestimate the risk of night works. Additionally, this association could be due to the problem of sparse data. Therefore, studies about the potential risks of fixed night shift for mental health should be continuously conducted, especially among healthy night workers.

In the conducted subgroup analysis on shift work, it was observed that there is a risk of suicidal ideation among both men (OR 1.24, 95% CI 1.08–1.42) and women (OR 1.34, 95% CI 1.17–1.55). Several meta-analyses have been conducted on the association between shift work and mental health problems, which exhibited an increased risk among women (68, 74). However, our findings revealed an elevated risk in both. Therefore, considering that the participants with various working conditions and taking into account gender division in labor markets, it is crucial to recognize the possibility of occupational factors affecting gender comparisons (75).

Furthermore, considering specific occupational groups, a sub-analysis shows that manufacturing workers exposed to shift work were at higher risk (OR 1.86, 95% CI 1.17–2.97) compared to general workers (OR 1.31, 95% CI 1.20–1.44). It is important to recognize the essential nature of shift work and long hours in industries such as manufacturing, healthcare, transport, and first response work (9). Therefore, promoting screening and interventions to reduce the potential risks in these industries is crucial.

Extensive research has been conducted on the association between long working hours, shift work, and mental health problems including depressive symptoms and suicidal ideation (56, 76–81). Long working hours and shift work have been shown to be risk factors that increase the risk of mental health problems such as depression, anxiety, alcohol abuse, and sleep disorders. These mental health problems are associated with an increased risk of suicidal ideation. Some studies discuss potential mechanisms underlying suicidal ideation and emphasize the importance of careful management (82). Previous studies further support this finding, showing a strong correlation between suicidal ideation or self-harm and the severity of depressive symptoms measured with the depression assessment tool, PHQ-9 (83). Furthermore, the fact that approximately 50% of individuals who die by suicide have mental health problems provides an explanation for this potential mechanism (84). According to the ideation-to-action framework of suicide theory, the prevention of suicidal ideation is critical to the reduction of suicide rates (85). Therefore, minimizing workers’ exposure to occupational factors that negatively impact mental health, and implementing legal sanctions and support to reduce long working hours are crucial steps in promoting workers’ mental well-being.

### Strengths and limitations

This study is particularly relevant because of the growing interest in working hours prompted by recent shifts in the labor market. This study provided a robust meta-analysis that thoroughly investigated the correlation between occupational factors, specifically long working hours and shift work, and suicidal ideation. This review adhered to the established PRISMA guidelines for systematic reviews, ensuring methodological rigor. The comprehensive literature review involved meticulous searches across multiple databases using comprehensive search terms. Additional measures were implemented to minimize bias, including a thorough examination of relevant articles’ reference lists.

This study has the following limitations. First, it is important to note that a significant portion of this study is focused on Asia, particularly Korea. This demonstrates that long working hours and shift work are the most significant policy and social issues in Korea. Specifically, it emphasizes the exceptionally long annual working hours and the lack of holidays compared to other OECD countries, which are prevalent issues in Korea (86). However, the sensitivity analysis revealed that the risk of suicidal ideation attributed to long working hours was statistically higher in countries other than Korea. Therefore, we cannot conclude that the estimate was overestimated solely due to publication bias in one country. Second, despite the comprehensive search process, publication bias may have affected the literature reviews and meta-analyses. This implies that the true effect size may be smaller than the initially estimated value, and the observed increases may no longer be statistically significant. Therefore, we corrected the publication bias using the trim-and-fill method; however, the effect size needs to be evaluated more carefully (35). It is crucial to consider the potential impact of publication bias in the interpretation of results and to assess the clinical significance of the observed effect sizes (87).

Third, the studies included in this analysis were mostly cross-sectional, with some using partial data from longitudinal studies. Although cross-sectional studies provide an initial exploration of the research questions, this design has certain limitations. In future studies, a longitudinal design should be used to better understand causal pathways and to account for the temporal nature of observed effects (88). In conclusion, while this study provides valuable insights, awareness of these limitations is essential for a nuanced interpretation of the findings and the guidance of future research.

Fourth, risk factors that influence suicidal ideation are not fully accounted for. In this study, only manufacturing and non-manufacturing industries were categorized as occupational factors. This is because we did not have enough numbers to synthesize detailed characteristics such as other industries and work characteristics (working years, work position, etc.) in the selected studies. In addition, although we synthesized the results by extracting the adjusted values of the variables considered as risk factors for suicidal ideation in the selected studies, it is possible that non-work factors such as family demands and commuting time were not sufficiently considered.

Finally, suicidal ideation was measured through self-report surveys, which may be influenced by self-report bias. Relying solely on these surveys also poses a risk, as participants might knowingly or unknowingly provide inaccurate information. However, adherence to standardized response criteria can help mitigate the potential impact of these biases (89).

### Concluding remarks

The association between occupational factors and suicide is linked to elevated levels of psychological issues and poor mental health. This study highlights the significant association between nonstandard working hours (long working hours or shift work) and suicidal ideation among workers, underscoring the importance of identifying modifiable risk factors that influence such ideation.

The proactive identification and management of psychosocial occupational risks are crucial for identifying high-risk individuals and providing timely treatment. Preventing the risk of suicidal ideation among workers requires multifaceted interventions, including legal measures, reporting systems for excessive work, workplace fines, flexible work patterns, adequate staffing, vacation provisions, and health programs that allow periodic risk assessments. Evidence-based interventions that address these risk factors can prevent the progression of suicidal ideation. It is also crucial to conduct further studies that demonstrate the interactions and temporal associations of risk factors while staying up to date with the latest research.

## Supplementary material

Supplementary material
