# Supplementary material for: The association between long working hours, shift work, and suicidal ideation: A systematic review and meta-analyses
Source: Scand J Work Environ Health. 2024 Sep 30;50(7):503–18. doi: 10.5271/sjweh.4182 (PMC11472300; doi:10.5271/sjweh.4182)
Supplement: Supplementary material [file SJWEH-50-503-S001.pdf]

The association between long working hours, shift work, and suicidal ideation: A systematic review and meta-analyses[1]

by Joungsue Kim, PhD candidate, Ria Kwon, PhD, Hyunok Yun, MPH, Ga-Young Lim, PhD, Kyung-Sook Woo, PhD, Inah Kim, PhD [2]

1. Supplementary material
2. Correspondence to: Inah Kim, Department of Occupational and Environmental Medicine, College of Medicine, Hanyang University, Seoul 04763, Korea. [E-mail: inahkim@hanyang.ac.kr]

Appendix A: Overview of inclusion and exclusion criteria

Include

1. Quantitative studies of any design, including cross-sectional studies, on the prevalence of suicidal ideation for the years 1970 to 2022
2. Studies of the working-age population (≥ 18 year)
3. Studies on populations residing in any Member States of WHO and/or ILO and any industrial sector or occupational group setting
4. Studies on the prevalence of suicidal ideation,
5. Studies published between 1970. 01. 01-2022. 09. 30 and English and Korean language

Exclude

1. Studies of unpaid domestic workers
2. Studies of youth and younger (< 18 years)
3. Studies with data only on years other than between 1970 and 2022
4. Qualitative and case studies, as well as non-original studies without quantitative data (e.g. letters, commentaries and perspectives)

Appendix B: Overview

Supplementary table S 1.

Pubmed			
			검색일 : 2022.10.26.
PECO	#	Query	Results
[P]<br>Long working hour<br>Shift work	1	work*[Title/Abstract]	1,888,937
	2	hour*[Title/Abstract]	627,401
	3	time*[Title/Abstract]	4,350,747
	4	shift*[Title/Abstract]	461,851
	5	night*[Title/Abstract]	97,889
	6	1 AND (2 OR 3 OR 4 OR 5)	465,827
	7	overtime*[Title/Abstract]	3,976
	8	overwork*[Title/Abstract]	1,103
	9	long work* hour*[Title/Abstract]	307
	10	6 OR 7 OR 8 OR 9	469,663
[O]<br>Suicide ideation	11	suicid*[Title/Abstract]	92,143
[P] and [O]	12	10 AND 11	1,857
	13	12 Filters: English, Korean, Humans, from 1970 - 2022/9/30	1,325

Supplementary table S 2.

Embase			
			검색일 : 2022.10.26.
PECO	#	Query	Results
[P]<br>Long working hour	1	overtime.mp.	6,808
	2	overwork.mp.	725
	3	exp working time/ or long working hour*.mp. or exp workload/	61,501
[P]<br>Shift work	4	exp shift worker/ or exp evening shift/ or exp night shift/ or exp shift work/ or shift*.mp. or exp night shift worker/	535,829
	5	1 or 2 or 3 or 4	599,257
[O]<br>Suicide ideation	6	exp suicidal ideation/ or suicid*.mp.	152,140
[P] and [O]	7	5 AND 6	1,648
	8	limit 8 to ((english or korean) and yr="1970 - 2022")	1,403

Supplementary table S 3.

CINAHL			
			date : 2022.11.01
PECO	#	Query	Results
[P] Long working hour Shift work	1	work*[Title]	161,601
	2	work*[Abstract]	464,658
	3	1 OR 2	551,726
	4	time*[Title]	102,596
	5	time*[Abstract]	751,854
	6	4 OR 5	815,892
	7	hour*[Title]	8,658
	8	hour*[Abstract]	119,285
	9	7 OR 8	124,911
	**10**	**3 AND (6 OR 9)**	**107,823**
	11	overtime*[Title]	815
	12	overtime*[Abstract]	1,634
	13	11 OR 12	2,317
	14	overwork*[Title]	176
	15	overwork*[Abstract]	431
	16	14 OR 15	582
	17	long work*[Title]	1,108
	18	long work*[Abstract]	4,611
	19	17 OR 18	5,363
	20	shift work*[Title]	1,525
	21	shift work*[Abstract]	4,603
	22	20 OR 21	5,380
	23	night work*[Title]	484
	24	night work*[Abstract]	1,346
	25	23 OR 24	1,617
	**26**	**10 OR 13 OR 16 OR 19 OR 22 OR 25**	**116,761**
[O] Suicide ideation	27	suicid*[Title]	24,983
	28	suicid*[Abstract]	30,172
	29	27 OR 28	39,472
[P] and [O]	**30**	**26 AND 29**	**732**
	31	Limiter - Publication date: 19700101-20220930; Language: English, Korean<br>Extension - Related words (synonyms, plural) Extended search; Equivalent subject application	715

Appendix C: Rate risk of bias

The risk of bias for each study included in the analysis underwent independent evaluation by four authors, organized into two groups. In instances of disparities, consensus was achieved through discussion or consultation.

The Navigation Guide methodology was employed to assess the risk of bias in each study contributing to the meta-analysis[1] [2]. The Navigation Guide utilizes nine domains for evaluation, namely: (i) source population representation; (ii) blinding; (iii) exposure assessment; (iv) outcome evaluation; (v) confounding; (vi) incomplete outcome data; (vii) reporting optional results; (viii) conflicts of interest; and (ix) other sources of bias. Ratings for risk of bias or confounding across all domains were categorized as "low", "probably low", "probably high", "High", or "N/A". (Appendix 1.2

These questions have also been modified from previous applications of the Navigation Guide, with edits intended so that answering "No" to each question aligns with a rating of "Low risk of bias", "Probably No" → "Probably low risk of bias", "Probably Yes" → "Probably high risk of bias" and "Yes" → "high risk of bias." 'Probably' refers to cases that are not directly stated in the paper, but can be confirmed indirectly. The criteria for evaluating bias are presented in the appendix.

---

1 Lam J, Sutton P, Padula A, Cabana M, Koustas E, Vesterinen H, et al. Applying the Navigation Guide Systematic review methodology case study# 6: association between formaldehyde exposure and asthma: a systematic review of the evidence: protocol. University of California at San Francisco, San Francisco, CA. 2016.

2 Higgins JP, Altman DG. Assessing risk of bias in included studies. Cochrane handbook for systematic reviews of interventions: Cochrane book series. 2008:187-241.

1. Domain 1. Are the study groups at risk of not representing their source populations in the manner that might introduce selection bias?

Bias	Authors' assessment of risk of bias	Support for assessment
1. Are the study groups at risk of not representing their source populations in the manner that might introduce selection bias?	***LOW***	-Study participants were recruited from the same population during the same time period, or<br>-Study participants are not all recruited from the same population, there is a consistent proportion of participants from each population in each study group
	PROBABLY LOW	There is insufficient information about participant selection to determine 'Low', but there is indirect evidence to suggest that participant recruitment and inclusion/exclusion criteria were consistent as described in the 'Low' judgment criteria.
	PROBABLY HIGH	There is insufficient information regarding participant selection to make a judgment of "high". However, there is indirect evidence indicating that participant recruitment or inclusion/exclusion criteria were inconsistent, as described in the criteria for a "high" judgment.
	HIGH	-Recruitment protocols or inclusion/exclusion criteria were applied differently between study groups.<br>-Study participants were recruited at different times.<br>-When study participants are recruited from different groups, the proportion of participants from each group in each study group is not uniform.<br>There was differential loss to follow-up between groups.<br>The reported refusal/non-response is not uniform across groups

2. Domain 2. Was knowledge of the exposure adequately prevented (i.e. blinded or masked) prevented during the study, potentially leading to subjective measurement of either exposure or outcome?

Bias	Authors' assessment of risk of bias	Support for assessment
2. Was knowledge of the exposure adequately prevented (i.e. blinded or masked) prevented during the study, potentially leading to subjective measurement of	***LOW***	-There was no blinding, but the review authors determined that outcome and outcome measures, as well as exposure and exposure measures, were unlikely to be affected by the lack of blinding.<br>-The blinding of key research participants l is guaranteed, and it is unlikely that the blinding was compromised.<br>-Some key study participants were not blinded, but the exposure and outcome assessments were blinded, and it is unlikely that bias will occur due to the non-blinding of other study personnel. This indicates a low risk of bias.

either exposure or outcome?	***PROBABLY LOW***	There is not enough information about blinding to determin e if the study has a low risk of bias. However, there is ind irect evidence that suggests the study was correctly blinded , as described in the criteria for determining a low risk of bias.
	PROBABLY HIGH	There is insufficient information regarding blinding to deter mine a high risk of bias. However, there is indirect eviden ce that suggests the study may not have been adequately bl inded, as described in the criteria for determining a high ri sk of bias.
	HIGH	- No blinding or incomplete blinding, and the outcome or outcome measurement or exposure and exposure measurem ent is likely to be influenced by lack of blinding (i.e., diffe rential outcome or exposure assessment<br>- When attempts to blind key research personnel are made, but the blinding is broken, leading to bias.<br>- When some key research personnel are not blinded, and t he lack of blinding in other research personnel is likely to introduce bias.

3. Domain 3. Were exposure assessment methods lacking accuracy?

**Bias**	**Authors' assessment of risk of bias**	**Support for assessment**
3. Were exposure assessment methods lacking accuracy?	***LOW***	There is a high level of confidence in the accuracy of expo sure assessment methods, especially those that have been v alidated and proven reliable in measuring the intended expo sure.<br>Definitions of exposure are consistently used, and exposure measurements are assessed based on exposure levels during the relevant reporting period. These measurements are repor ted directly, rather than through a proxy.
	PROBABLY LOW	There is not enough information provided about the specifi c exposure assessment method to determine if the risk of bi as is low, there is indirect evidence that suggests the metho d was robust, as outlined in the criteria for assessing bias.
	PROBABLY HIGH	There is insufficient information regarding the exposure ass essment method to determine a high risk of bias. However, there is indirect evidence that suggests the method describe d does not meet the criteria for a robust assessment.
	HIGH	The reviewer identifies that there is a high risk of exposure misclassification if one of the conditions below is met:<br>- There is low confidence in the accuracy of the exposure assessment method.<br>- There is uncertainty about how the exposure information was obtained.

4. Domain 4. Were outcome assessment methods lacking accuracy?

Bias	Authors' assessment of risk of bias	Support for assessment
4. Were outcome assessment methods lacking accuracy?	***LOW***	The outcome (suicidal ideation) was assessed and defined c onsistently for all study participants using valid and reliable scales. The accuracy of the outcome assessment methods s hould be evaluated based on the specific details provided in each study.
	PROBABLY LOW	There is insufficient information about the methods, there is indirect evidence suggesting that the methods were robust, as described in the low risk of bias criteria.
	PROBABLY HIGH	There is not enough information about the outcome assessm ent method to make a clear judgment about the risk of bias . However, there is indirect evidence indicating that the met hod described does not meet the criteria for a high risk of bias.
	HIGH	The reviewer determines that the results are at a high risk of being misclassified, and one of the following applies:<br>· There is low confidence in the accuracy of the outcome a ssessment method.<br>· Less established or less direct outcome measures have not been validated and are suspected of introducing bias that a ffects outcome assessments.<br>· It is unclear how the result information was obtained.

5. Domain 5. Was potential confounding inadequately incorporated?

A comprehensive list of important potential confounders was generated by the review authors before beginning the study screening process. This list was based on expert opinion and knowledge gathered from the literature.

Tier I: Important confounders : sex, age, socioeconomic position

Tier II: Other potentially important confounders and effect modifiers: Type of industry type, type of occupation, country

Bias	Authors' assessment of risk of bias	Support for assessment
5. Was potential confounding inadequately incorporated?	***LOW***	The study appropriately assessed and accounted for all important confounding factors (such as gender, age, socioeconomic status) through various methods like matching, stratifying, excluding certain groups, or statistically controlling for them. It was reported that some confounding factors were omitted because they did not significantly affect the results. The determination of specific confounders may be based on studies included in the full review.<br><br>The study adequately assessed and accounted for other potentially important confounders (such as industry type, occupation type, country) using appropriate statistical techniques. If these confounders were included, it must have been assessed and reported as missing because they did not have a significant effect on the study. The study must have also measured important potential confounding factors consistently across study groups using valid and reliable methods, or confirmed them through sensitivity analysis to minimize the impact of measurement errors.
	PROBABLY LOW	These studies adequately accounted for most (but not all) of the important confounding factors, such as gender, age, and socioeconomic status, or used appropriate statistical techniques.<br><br>They reported that other potentially important confounders, such as industry type, job type, and country, were included using appropriate statistical techniques, or that the inclusion of these confounders did not significantly impact the results and were therefore evaluated and omitted.<br><br>Moreover, it is not expected that these confounders will cause significant bias.
	PROBABLY HIGH	The study should consider some, but not all, of the significant confounding factors (such as gender, age, socioeconomic status) and other potentially relevant confounding factors (such as industry type, occupation, and country). Additionally, the study should not rely on questionable statistical techniques to adjust for confounders.
	HIGH	The study did not consider or evaluate several significant confounding factors, such as gender, age, and socioeconomic status. It also failed to consider or evaluate other potentially important factors, including industry type, job type, and country. Furthermore, the study did not appropriately measure or analyze these factors across the different study groups.

6. Domain 6. Were incomplete outcome data inadequately addressed?

Bias	Authors' assessment of risk of bias	Support for assessment
6. Were incomplete outcome data inadequately addressed?	***LOW***	Participants were followed for a sufficient duration to collec t outcome measures. The following conditions were met:<br><br>- No outcome data are missing.<br>- The data missing between exposure groups are similar, an d any resultant data loss or omission is due to numerical ba lance between the groups.<br>- For dichotomous outcome data, the proportion of missing outcomes compared to the observed event risk is not signifi cant enough to impact the estimates of the intervention effe ct.<br>- For continuous outcome data, the missing outcome's plaus ible effect size (mean difference or standardized mean differ ence) is not large enough to have a meaningful impact on t he observed effect size.<br>- Any missing data were estimated using appropriate metho ds.
	PROBABLY LOW	There is insufficient information about incomplete outcome data to determine a low risk of bias, there is indirect eviden ce suggesting that the handling of incomplete outcome data was appropriate according to the criteria for determining a l ow risk of bias.
	PROBABLY HIGH	There is insufficient information regarding incomplete outco me data to establish a high risk of bias, there is indirect evi dence indicating that the handling of incomplete outcome d ata may not have been in accordance with the criteria for d etermining a high risk of bias.
	HIGH	Participants were not followed for a sufficient duration to g ather outcome measures, unless one of the following conditi ons is met:<br><br>- The reason for missing outcome data is likely to be relate d to the actual outcome and is associated with a numerical imbalance between exposure groups or reasons for missing data.<br>- For dichotomous outcome data, the proportion of missing outcomes compared to observed event risk is significant eno ugh to result in biologically relevant bias in estimates of th e intervention effect.<br>- For continuous outcome data, the plausible effect size (me an difference or standardized mean difference) among missi ng outcomes is large enough to cause biologically relevant bias in the observed effect size.<br>- There is a potentially inappropriate application of substitut ion.

7. Domain 7. Does the study appear to have selective outcome reporting?

Bias	Authors' assessment of risk of bias	Support for assessment
7. Was knowledge of the exposure adequately prevented (i.e. blinded or masked) prevented during the study, potentially leading to subjective measurement of either exposure or outcome?	***LOW***	Of all the prespecified (primary and secondary) outcomes m entioned in the study protocol, methods, abstract, and/or intr oduction, the review only focused on those outcomes that w ere reported in a prespecified manner.
	PROBABLY LOW	There is not enough information about selective reporting of outcomes to determine whether the study is at a low risk o f bias. However, there is indirect evidence suggesting that t he study did not have selective reporting, as described in th e criteria for determining a low risk of bias.
	PROBABLY HIGH	There is insufficient information about selective reporting of outcomes to determine whether the study is at a high risk of bias. However, based on the criteria for determining a hi gh risk of bias, there is indirect evidence suggesting that th e study does not exhibit selective reporting.
	HIGH	- The study failed to report all pre-specified primary outco mes, as outlined in the protocol, methods, abstract, and/or i ntroduction.<br>- One or more primary outcomes were reported using a me asure, analysis method, or subset of data (e.g., subscale) tha t was not pre-specified.<br>- One or more of the main outcomes reported were not pre-specified, and no clear rationale for reporting them was pro vided, such as unexpected effects.<br>- There was incomplete reporting of one or more outcomes of interest.

8. Domain 8. Did the study receive any support from a company, study author, or other entity having a financial interest in any of the exposures studied?

Bias	Authors' assessment of risk of bias	Support for assessment
8. Was knowledge of the exposure adequately prevented (i.e. blinded or masked) prevented during the study, potentially leading to subjective measurement of either exposure or outcome?	***LOW***	The study was not funded by any company, study author, o r other entity with a financial interest in the study results. Here are some further details:<br>- Funding sources are limited to government, non-profit org anizations, or academic grants funded by governments, foun dations, and/or non-profit organizations.<br>- Chemicals or other treatments used in the study were pur chased from a supplier.<br>- Company employees are not mentioned in the Acknowled gments section.<br>- The authors are not employees of any company with a fin ancial interest in the research results.<br>- No company with a financial interest in the study was inv olved in the design, conduct, analysis, or reporting of the st

		udy, and the authors had full access to the data.<br>- If the study author makes a claim denying a conflict of in terest.<br>- The study authors are not associated with any company w ith a financial interest, and there is no reason to believe a c onflict of interest exists.<br>- All study authors are affiliated with government agencies, and they are prohibited from participating in projects where there is a conflict of interest or the appearance of a confli ct of interest.
	PROBABLY LOW	There is not enough information to determine if the risk of bias is low. However, this implies that the study was not fu nded by a company, study author, or any other entity with a financial interest in the study's results, as stated in the cri teria for assessing a low risk of bias. If there is indirect evi dence that
	PROBABLY HIGH	There is insufficient information to permit a judgment of hi gh risk of bias, but there is indirect evidence that suggests the study was not free of support from a company, study a uthor, or other entity having a financial interest in the outc ome of the study, as described by the criteria for a judgme nt of high risk of bias.
	HIGH	The study was funded by a company, study author, or other entity with a financial interest in the study results. Exampl es of support include:<br>- Research funding;<br>- Writing services;<br>- If the authors/employees who participated in the study are employees of the company or affiliated with a company in which they have other financial interests;<br>- If the company was involved in the design, conduct, anal ysis, or reporting of the study;<br>- If the study author claims a conflict of interest.

9. Domain 9. Did the study appear to have other problems that could put it at a risk of bias?

Bias	Authors' assessment of risk of bias	Support for assessment
9. Was knowledge of the exposure adequately prevented (i.e. blinded or masked) prevented during the study, potentially leading to subjective measurement of	***LOW***	There do not appear to be any other sources of bias in the study.
	PROBABLY LOW	There is insufficient information to determine that the risk o f bias is low. However, there is indirect evidence suggesting that there are no other threats to the validity of the study.
	PROBABLY HIGH	There is insufficient information to determine a high risk of bias, but there is indirect evidence to suggest that there are no other threats to the validity of the study, as described i n the criteria for determining a high risk of bias.

either exposure or outcome?	***HIGH***	There is at least one significant risk of bias. For example, research:<br>- There is a potential source of bias related to the specific study design used, or<br>- The study is prematurely discontinued due to some procedure (including formal discontinuation rules) that relies on the data, or<br>- The conduct of the study was affected by intermediate results (e.g. recruiting additional participants from subgroups shown to have a greater or lesser effect), or<br>- An allegation is made that there has been fraud, or<br>- There are other problems.

Study/Navigation Guide risk of bias ratings																
Exposure	Long working hours															
Navigation Guide (Woodruff and Sutton 2014) Risk of bias domain	Tyssen (2001)	Al-Maskari (2011)	Langhinrichsen-Rohling (2011)	Kim (2012)	Choi (2018)	Liu (2019)	Park (2020)	Petrie (2020)	Choi (2021)	Kim (2021)	Malone (2021)	Xu (2021)	Ishikawa (2022A)	Ishikawa (2022B)	Lee (2022)	Li (2022)
1. Are the study groups at risk of not representing their source populations in a manner that might introduce **selection bias**?	Probably low	Probably high	Low	Low	Low	Probably low	Low	Probably	Low	Low	Probably low	Probably low	Probably low	Probably low	Low	Low
2. Was knowledge of the exposure adequately prevented (i.e. **blinded** or masked) prevented during the study potentially leading to subjective	Probably low	Probably high	Low	Low	Probably low	Low	Low	Low	Low	Low	Probably	Low	Probably	Probably low	Low	Low
3. Were exposure assessment methods lacking accuracy?	Low	Low	Low	Low	Probably low	Probably low	Low	Probably low	Low	Low	Probably high	Probably low	Probably high	Probably low	Low	Probably low
4. Were outcome assessment methods lacking accuracy?	Low	Probably low	Low	Low	Low	Low	Low	Low	Low	Low	Low	Low	Low	Low	Low	Low
5. Was potential confounding inadequately incorporated?	Probably low	Low	Probably low	Low	Probably low	Probably low	Probably low	Low	Low	Low	Low	Low	Low	Low	Low	Probably low
6. Were incomplete outcome data inadequately addressed?	Probably low	Probably low	Low	Low	Low	Low	Low	Probably low	Low	Low	Low	Probably low	Probably high	Probably low	Low	Probably low
7. Does the study appear to have selective outcome reporting?	Low	Probably low	Low	Low	Low	Probably low	Low	Probably low	Low	Low	Low	Low	Probably low	Low	Low	Low
8. Did the study receive any support from a company, study author, or other entity having a financial interest in any of the exposures studied?	Low	Low	Low	Low	Low	Low	Low	Low	Low	Low	Low	Low	Low	Low	Low	Low
9. Did the study appear to have other problems that could put it at a risk of bias?	Probably low	Probably low	Low	Probably low	Probably low	Probably low	Probably low	Probably low	Low	Low	Probably low	Low	Probably low	Probably low	Probably low	Probably low

Study/Navigation Guide risk of bias ratings																	
Exposure	Shift work								Both								
Navigation Guide (Woodruff and Sutton 2014) Risk of bias domain	Violanti (2008)	Takusari (2011)	Kanget (2017)	Kim (2019)	Park (2019)	Ahn (2020)	Son & Lee (2021)	Kim (2022)	Park (2022)	Yoon (2015)	Yoon (2015)	Chin (2018)	Kim (2020)	Lin (2020)	Niedhammer (2020)	Bryant-Lees (2021)	Han (2021)
1. Are the study groups at risk of not representing their source populations in a manner that might introduce **selection bias**?	Low	Probably low	Probably low	Low	Low	Probably low	Probably low	Low	Low	Low	Low	Probably high	Probably low	Probably low	Low	Probably low	Low
2. Was knowledge of the exposure adequately prevented (i.e. **blinded** or masked) prevented during the study potentially leading to subjective	Low	Low	Low	Low	Probably low	Low	Probably low	Low	Low	Low	Low	Low	Low	Probably low	Low	Low	Low
3. Were exposure assessment methods lacking accuracy?	Low	Low	Low	Low	Low	Probably low	Low	Probably low	Low	Low	Probably low	Probably low	Low	Low	Low	Low	Low
4. Were outcome assessment methods lacking accuracy?	Low	Low	Low	Low	Low	Low	Low	Low	Low	Low	Low	Low	Low	Low	Low	Low	Low
5. Was potential confounding inadequately incorporated?	Probably low	Low	Low	Low	Low	Low	Probably low	Low	Probably low	Low	Low	Probably low	Low	Low	Low	Probably low	Low
6. Were incomplete outcome data inadequately addressed?	Low	Probably low	Low	Low	Low	Low	Low	Low	Low	Low	Low	Low	Low	Low	Low	Probably low	Low
7. Does the study appear to have selective outcome reporting?	Low	Low	Low	Low	Low	Low	Low	Low	Low	Low	Low	Low	Low	Low	Low	Probably low	Low
8. Did the study receive any support from a company, study author, or other entity having a financial interest in any of the exposures studied?	Low	Low	Probably low	Low	Low	Low	Low	Low	Low	Low	Low	Low	Low	Low	Low	Probably low	Low
9. Did the study appear to have other problems that could put it at a risk of bias?	Probably low	Low	Low	Low	Probably low	Low	Probably low	Low	Probably low	Low	Low	Probably low	Low	Low	Low	Probably high	Low

Supplementary Figure C 1. Summary Risk of bias

Appendix D: Instructions for grading the quality of evidence

We attempted to GRADE to assess the overall evidence on the impact of long working hours and shift work on suicidal ideation. The GRADE evaluation took five things into considerations: (i) risk of bias; (ii) inconsistency; (iii) indirectness; (iv) imprecision; and (v) publication bias[3,4].

In case of risk of bias, the results evaluated through the Navigation Guide quality of evidence assessment tool were used. The Navigation tools are rated as low, probably low, probably high, and high in 9 items. For each document, if there were 0 Probably High, it was evaluated as good, if there was 1, it was evaluated as fair, and if there were 2, it was evaluated as poor. Among the articles included in the evaluation, if the ratio of good was more than 70%, it was evaluated as not serious, if the ratio of good was 40-70%, it was evaluated as serious, and if it was less than 40%, it was evaluated as very serious.

In case of inconsistency, I^2 statistics that verify statistical heterogeneity were used. If I^2 of less than 40 was evaluated as not serious, if I^2 of 40-60 was evaluated as serious, and if I^2 of more than 60 was evaluated as very serious.

In case of indirectness, most of the included studies were assessed as serious because they could not be described as having sufficient outcome timeframe.

In case of imprecision, sample size and the number of included studies were used. If the number of subjects included was more than 300, it was evaluated as not serious, if it included 100-300 subjects, it was evaluated as serious, and if it included less than 100 subjects, it was evaluated as very serious. Even if the number of subjects was more than 300, if the number of studies included was less than 5, it was evaluated as serious.

---

[3] Zhang, Y., Akl, E. A., & Schünemann, H. J. (2019). Using systematic reviews in guideline development: the GRADE approach. Research synthesis methods, 10(3), 312-329.

[4] Framework for Application of GRADE in CCS Guideline and Position Statement Development, https://ccs.ca/app/uploads/2021/07/CCS_GRADE_Framework_April2020.pdf

In case of publication bias, the Egger test results were used. If there appears to be a significant relationship between the standard error and the effect size, publication bias is assessed as being strongly suspected. In the egger test, if the p-value is over 0.05, it is assessed that there is no publication bias. If the number of studies is less than 10, the egger test cannot be performed and is evaluated as undetected.

Supplementary table S 4. Summary of finding: Effect of exposure to long working hours on suicidal ideation among workers

**Certainty assessment**							**Summary of findings**		**Certainty**	**Importance**
**№ of studies**	**Study design**	**Risk of bias**	**Inconsistency**	**Indirectness**	**Imprecision**	**Publication bias**	**OR (95%CI)**	**I^2(%)**		
**Total population (Regular time working vs Long time working)**										
16	Longitudinal n=2, cross-sectional n=14	Not serious<br>- 'good': 13(81.3%)<br>- 'fair': 1(6.3%)<br>- 'poor': 2(12.5%)	Very serious*	Serious	Not serious<br>- Total sample number: 131,673	Strongly suspected ++	1.457 (1.279–1.660)	70.6	⊕○○○ Very low	Critical
**Sub population (Standard working hours per week vs other working hours)**										
**Working hours (≤ 40 vs 41 - 48)**										
6	Cross-sectional n=6	Not serious<br>- 'good': 6(100%)	Not serious	Serious	Not serious<br>-Total sample number: 81,152	Undetected+*+*	1.004 (0.920–1.095)	0	⊕⊕⊕○ Moderate	Critical
**Working hours (≤ 40 vs 49 - 54)**										
3	Cross-sectional n=2	Not serious<br>- 'good': 3(100%)	Very serious*	Serious	Serious<br>-Total sample number: 23,774	Undetected+*+*	1.002 (0.861–2.485)	69.7	⊕○○○ Very low	Critical
**Working hours (≤ 40 vs ≥ 55)**										
7	Cross-sectional n=7	Not serious<br>- 'good': 6(85.7%)<br>- 'poor': 1(14.3%)	Very serious*	Serious	Not serious<br>-Total sample number: 85,458	Undetected+*+*	1.912 (1.471–2.485)	69.3	⊕⊕○○ Low	Critical
**Sub population (Maximum working hours per week vs other working hours)**										
**Working hours (≤ 48 vs >48)**										
9	Cross-sectional n=9	Not serious<br>- 'good': 8(88.9%)<br>- 'poor': 1(11.1%)	Very serious*	Serious	Not serious<br>-Total sample number: 106,611	Undetected+*+*	1.621 (1.338–1.964)	73.5	⊕⊕○○ Low	Critical

*: The I^2 value was over 60, indicating very high heterogeneity in the literature.
++: In the Egger test, there is a significant relationship between standard error and effect size.
+*+*: Risk assessment of publication bias was not performed as the number of articles included was less than 10.

Supplementary table S 5. Summary of finding: Effect of exposure to shift work on suicidal ideation among workers

Certainty assessment							Summary of findings		Certainty	Importance
№ of studies	Study design	Risk of bias	Inconsistency	Indirectness	Imprecision	Publication bias	OR (95%CI)	I^2(%)		
**Total population (Day or regular vs Shift or Night)**										
12	Cross-sectional n=12	Not serious<br>- ‘good’: 12(100%)	Very serious*	Serious	Not serious<br>- Total sample number: 229,337	None<br>- p-value: 0.052	1.316<br>(1.192–1.453)	64.1	⊕⊕◯◯<br>Low	Critical
**Type of Shift (Fixed Evening)**										
2	Cross-sectional n=2	Not serious<br>- ‘good’: 2(100%)	Very serious*	Serious	Serious<br>- Total sample number: 42,843	Undetected+*+*	1.247<br>(0.887–1.753)	68.5	⊕◯◯◯<br>Very low	Critical
**Type of Shift (Fixed Night)**										
2	Cross-sectional n=2	Not serious<br>- ‘good’: 2(100%)	Very serious*	Serious	Serious<br>- Total sample number: 42,288	Undetected+*+*	1.370<br>(1.025–1.830)	66.1	⊕◯◯◯<br>Very low	Critical
**Type of Shift (Shift work)**										
11	Cross-sectional n=10	Not serious<br>- ‘good’: 11(100%)	Very serious*	Serious	Not serious<br>- Total sample number: 219,531	None<br>- p-value: 0.058	1.326<br>(1.177–1.494)	65.0	⊕⊕◯◯<br>Low	Critical

*: The I^2 value was over 60, indicating very high heterogeneity in the literature.
++: In the Egger test, there is a significant relationship between standard error and effect size.
+*+*: Risk assessment of publication bias was not performed as the number of articles included was less than 10.

Appendix E: Preferred Reporting Items for Systematic reviews and Meta-Analyses extension for Scoping Reviews (PRISMA-ScR) Checklist

Supplementary table S 6. Preferred Reporting Items for Systematic reviews and Meta-Analyses extension for Scoping Reviews (PRISMA-ScR) Checklist

Section and Topic	Item #	Checklist item	Location where item is reported
**TITLE**			
Title	1	Identify the report as a systematic review.	p1
**ABSTRACT**			
Abstract	2	Provide a structured summary including, as applicable: background; objectives; data sources; study eligibility criteria, participants, and interventions; study appraisal and synthesis methods; results; limitations; conclusions and implications of key findings; systematic review registration number.	P1
**INTRODUCTION**			
Rationale	3	Describe the rationale for the review in the context of existing knowledge.	P2
Objectives	4	Provide an explicit statement of the objective(s) or question(s) the review addresses.	P2-3
**METHODS**			
Eligibility criteria	5	Specify the inclusion and exclusion criteria for the review and how studies were grouped for the syntheses.	P4<br>Appendix A
Information sources	6	Specify all databases, registers, websites, organisations, reference lists and other sources searched or consulted to identify studies. Specify the date when each source was last searched or consulted.	P3-4<br>Appendix B
Search strategy	7	Present the full search strategies for all databases, registers and websites, including any filters and limits used.	Appendix B
Selection process	8	Specify the methods used to decide whether a study met the inclusion criteria of the review, including how many reviewers screened each record and each report retrieved, whether they worked independently, and if applicable, details of automation tools used in the process.	P4
Data collection process	9	Specify the methods used to collect data from reports, including how many reviewers collected data from each report, whether they worked independently, any processes for obtaining or confirming data from study investigators, and if applicable, details of automation tools used in the process.	P4
Data items	10a	List and define all outcomes for which data were sought. Specify whether all results that were compatible with each outcome domain in each study were sought (e.g. for all measures, time points, analyses), and if not, the methods used to decide which results to collect.	P4-5
	10b	List and define all other variables for which data were sought (e.g. participant and intervention characteristics, funding sources). Describe any assumptions made about any missing or unclear information.	p5-6
Study risk of bias assessment	11	Specify the methods used to assess risk of bias in the included studies, including details of the tool(s) used, how many reviewers assessed each study and whether they worked independently, and if applicable, details of automation tools used in the process.	P3<br>Appendix C

Section and Topic	Item #	Checklist item	Location where item is reported
Effect measures	12	Specify for each outcome the effect measure(s) (e.g. risk ratio, mean difference) used in the synthesis or presentation of results.	P5-6
Synthesis methods	13a	Describe the processes used to decide which studies were eligible for each synthesis (e.g. tabulating the study intervention characteristics and comparing against the planned groups for each synthesis (item #5)).	P5<br>Appendix A
	13b	Describe any methods required to prepare the data for presentation or synthesis, such as handling of missing summary statistics, or data conversions.	P5-6
	13c	Describe any methods used to tabulate or visually display results of individual studies and syntheses.	P5-6
	13d	Describe any methods used to synthesize results and provide a rationale for the choice(s). If meta-analysis was performed, describe the model(s), method(s) to identify the presence and extent of statistical heterogeneity, and software package(s) used.	P5-6
	13e	Describe any methods used to explore possible causes of heterogeneity among study results (e.g. subgroup analysis, meta-regression).	P5
	13f	Describe any sensitivity analyses conducted to assess robustness of the synthesized results.	P6
Reporting bias assessment	14	Describe any methods used to assess risk of bias due to missing results in a synthesis (arising from reporting biases).	Appendix C
Certainty assessment	15	Describe any methods used to assess certainty (or confidence) in the body of evidence for an outcome.	Appendix D
**RESULTS**			
Study selection	16a	Describe the results of the search and selection process, from the number of records identified in the search to the number of studies included in the review, ideally using a flow diagram.	P7<br>Figure 1
	16b	Cite studies that might appear to meet the inclusion criteria, but which were excluded, and explain why they were excluded.	P7
Study characteristics	17	Cite each included study and present its characteristics.	P7, Table 1
Risk of bias in studies	18	Present assessments of risk of bias for each included study.	P35<br>Appendix C
Results of individual studies	19	For all outcomes, present, for each study: (a) summary statistics for each group (where appropriate) and (b) an effect estimate and its precision (e.g. confidence/credible interval), ideally using structured tables or plots.	Appendix F
Results of syntheses	20a	For each synthesis, briefly summarise the characteristics and risk of bias among contributing studies.	P7-8
	20b	Present results of all statistical syntheses conducted. If meta-analysis was done, present for each the summary estimate and its precision (e.g. confidence/credible interval) and measures of statistical heterogeneity. If comparing groups, describe the direction of the effect.	Figure 2, 3
	20c	Present results of all investigations of possible causes of heterogeneity among study results.	P9, Figure 4

Section and Topic	Item #	Checklist item	Location where item is reported
	20d	Present results of all sensitivity analyses conducted to assess the robustness of the synthesized results.	P9
Reporting biases	21	Present assessments of risk of bias due to missing results (arising from reporting biases) for each synthesis assessed.	Appendix C
Certainty of evidence	22	Present assessments of certainty (or confidence) in the body of evidence for each outcome assessed.	Appendix D
**DISCUSSION**			
Discussion	23a	Provide a general interpretation of the results in the context of other evidence.	p 9-12
	23b	Discuss any limitations of the evidence included in the review.	p 13-14
	23c	Discuss any limitations of the review processes used.	p 13-14
	23d	Discuss implications of the results for practice, policy, and future research.	p 15
**OTHER INFORMATION**			
Registration and protocol	24a	Provide registration information for the review, including register name and registration number, or state that the review was not registered.	N/A
	24b	Indicate where the review protocol can be accessed, or state that a protocol was not prepared.	N/A
	24c	Describe and explain any amendments to information provided at registration or in the protocol.	N/A
Support	25	Describe sources of financial or non-financial support for the review, and the role of the funders or sponsors in the review.	p 16
Competing interests	26	Declare any competing interests of review authors.	p 14
Availability of data, code and other materials	27	Report which of the following are publicly available and where they can be found: template data collection forms; data extracted from included studies; data used for all analyses; analytic code; any other materials used in the review.	N/A

From: Page MJ, McKenzie JE, Bossuyt PM, Boutron I, Hoffmann TC, Mulrow CD, et al. The PRISMA 2020 statement: an updated guideline for reporting systematic reviews. BMJ 2021;372:n71. doi: 10.1136/bmj.n71

For more information, visit: http://www.prisma-statement.org/

Appendix F: Results of individual studies

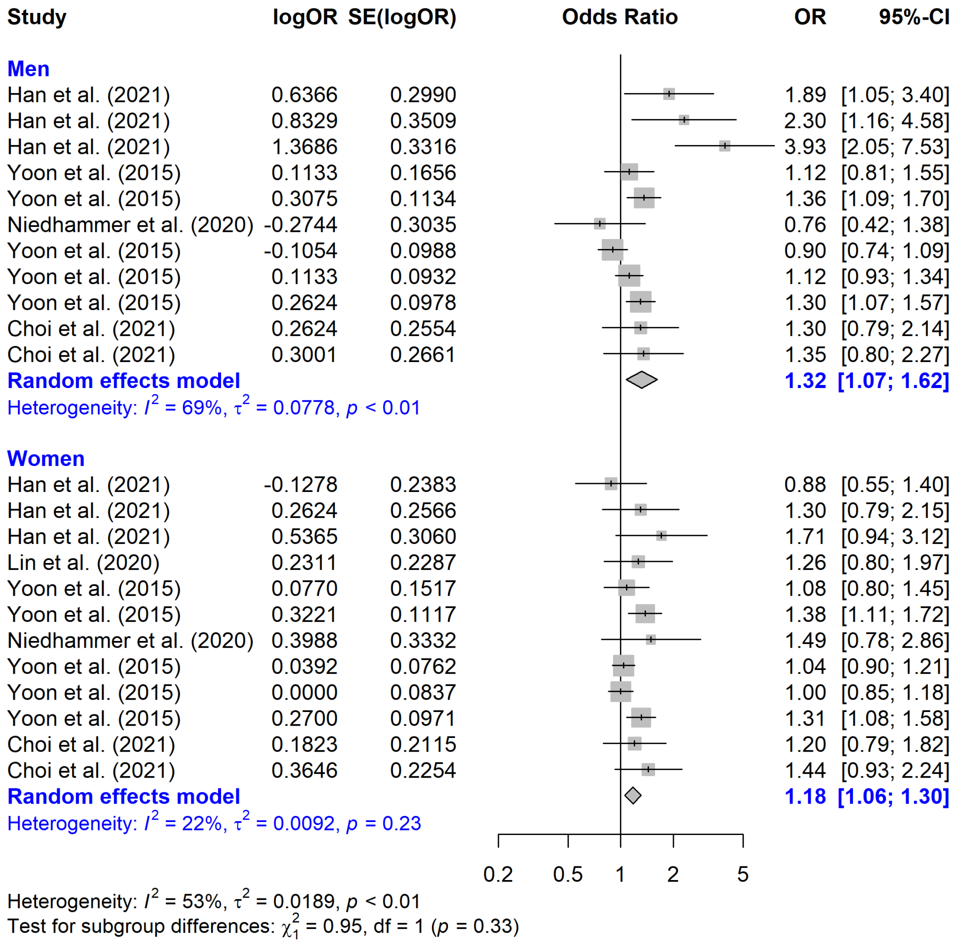

Supplementary Figure F1. Meta-analysis of gender differences in the association between long working hours and suicidal ideation

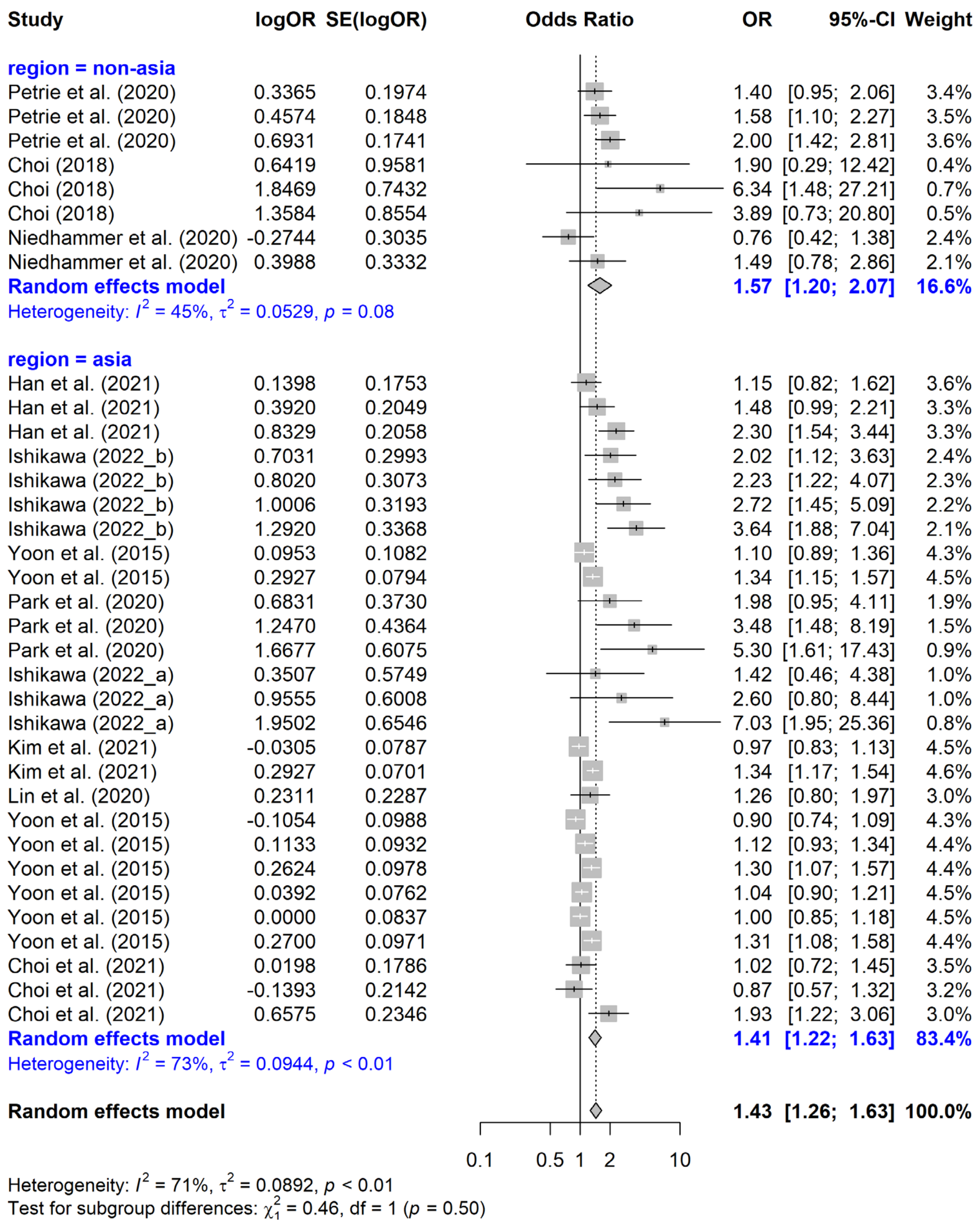

Supplementary Figure F2. Meta-analysis of region differences in the association between long working hours and suicidal ideation

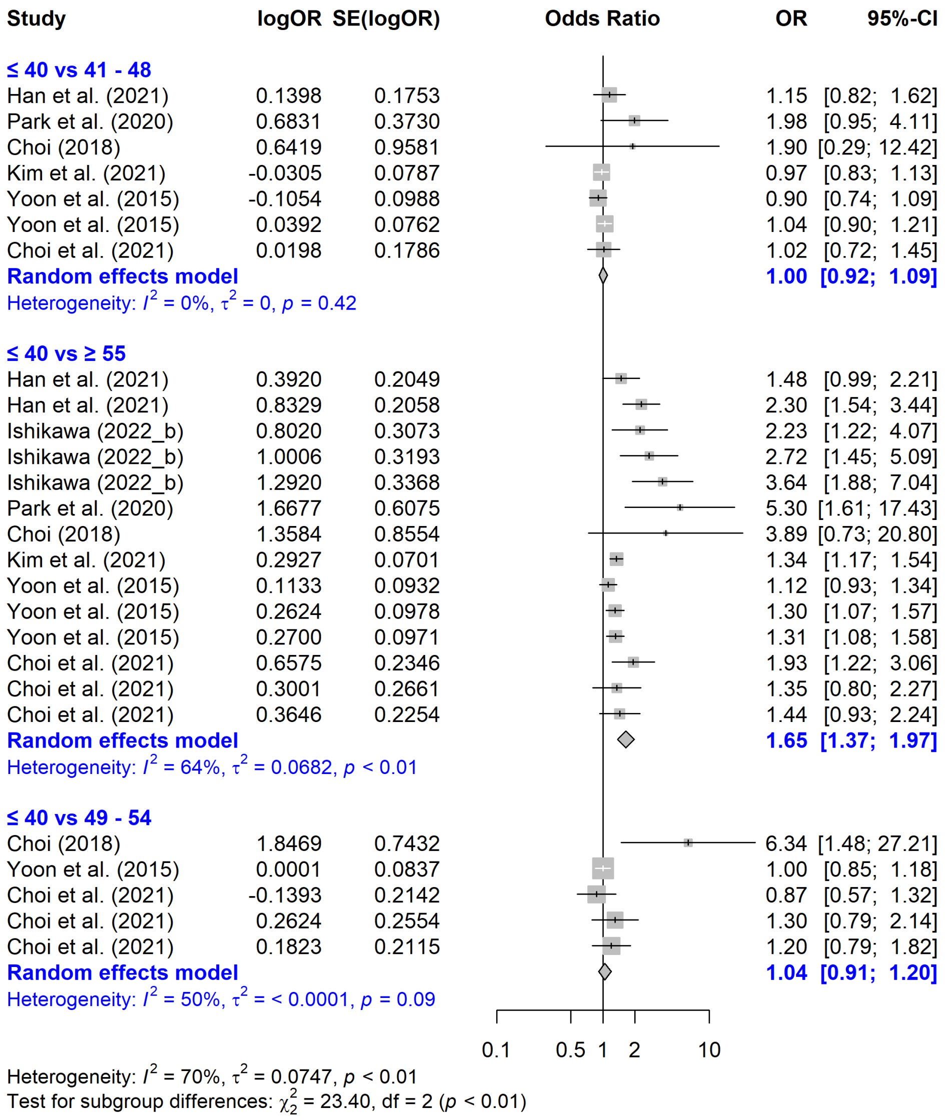

Supplementary Figure F3. Meta-analysis of differences in suicidal ideation by work hours (based on 40 hours)

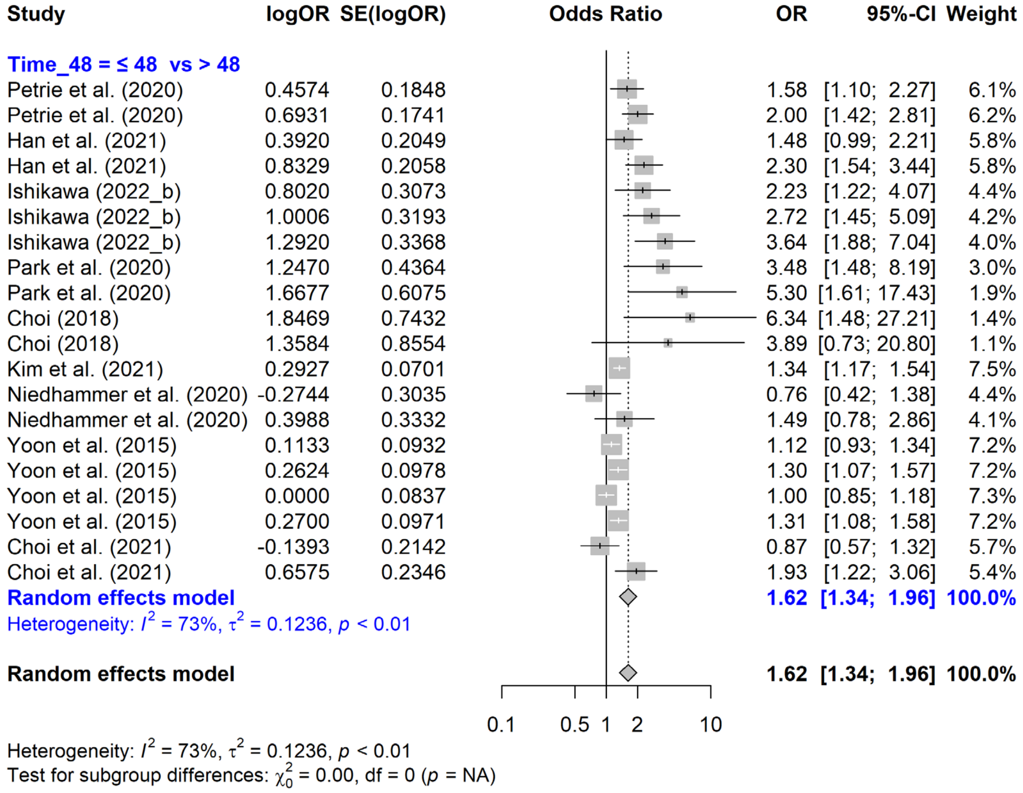

Supplementary Figure F4. Meta-analysis of differences in suicidal ideation by work hours (based on 48 hours)

Supplementary table S 7. Non-adjusted OR between Long working hours and suicide Ideation

	OR	95% CI	N_{OR}	I^2(%)
Overall analysis	1.438	1.212 - 1.706	22	89.8
Sub-group				
Gender				
Men only	1.043	0.848 - 1.283	5	90.2
Women only	1.394	1.044 - 1.861	7	90.9
Geographic region				
Asia	1.595	1.236 - 2.059	13	92
Non-Asia	1.276	1.037 - 1.570	9	84.2
Working hours (Standard working hours per week)				
≤ 40 vs 41 - 48	1.067	0.689 - 1.652	3	78.7
≤ 40 vs 49 - 54	-	-	-	-
≤ 40 vs ≥ 55	2.19	1.403 - 3.417	4	57.9
Working hours (Maximum working hours per week)				
≤ 40 vs > 40	1.56	1.165 - 2.083	12	90.6
≤ 48 vs > 48	1.438	1.001 - 2.065	7	86.4
Type of job				
Doctor	1.514	1.058 - 2.165	4	90.9
Other	1.423	1.165 - 1.739	18	90

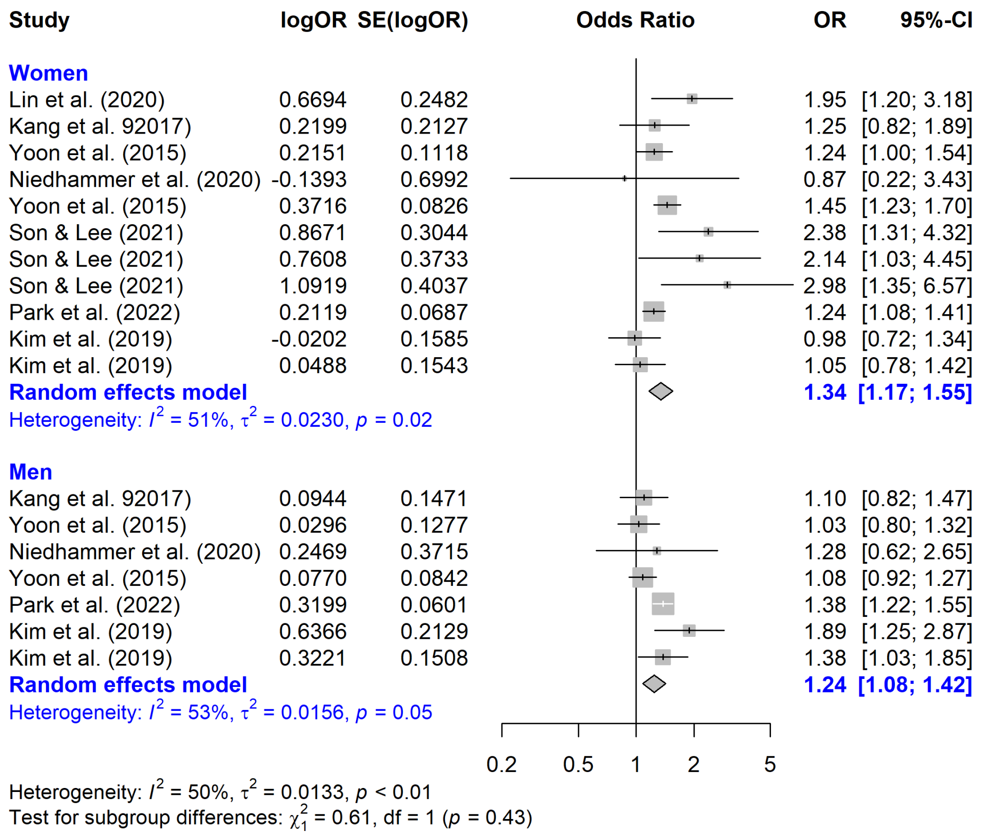

Supplementary Figure F5. Meta-analysis of gender differences in the association between shift work and suicidal ideation

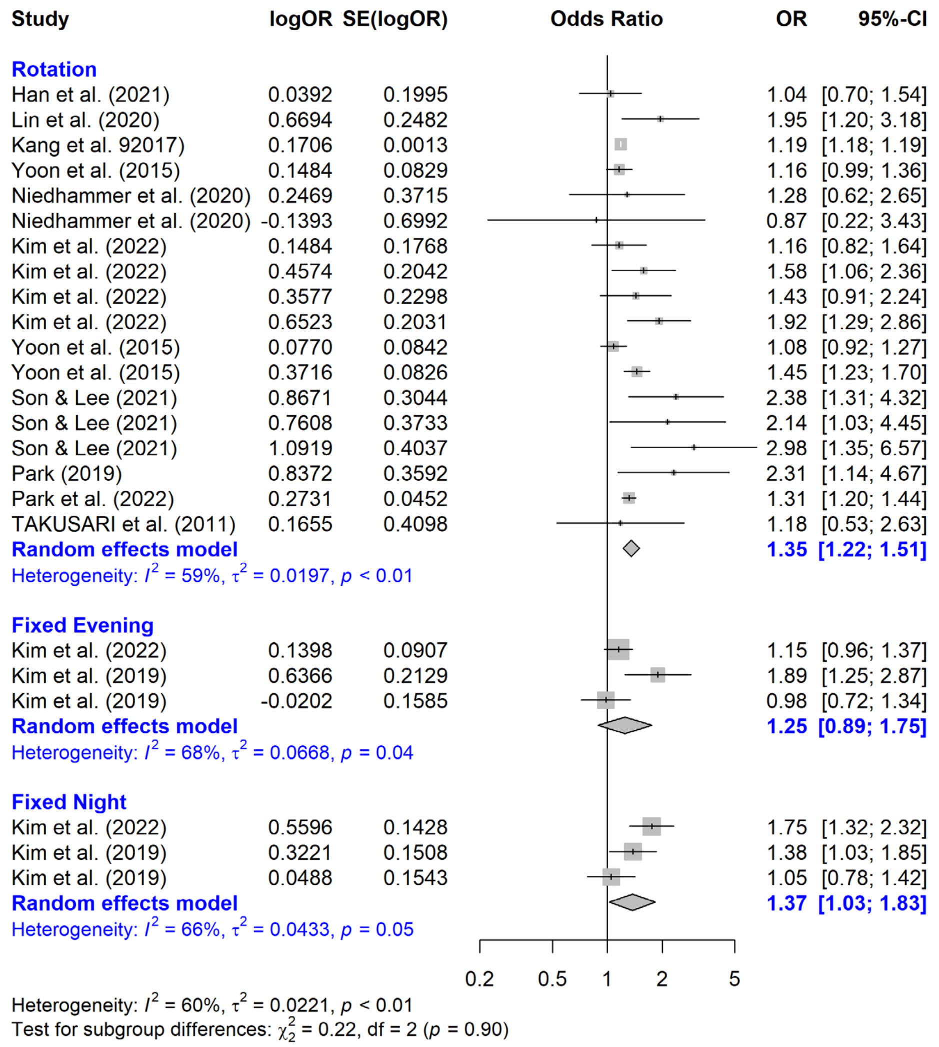

Supplementary Figure F6. Meta-analysis of differences in suicidal ideation by work type

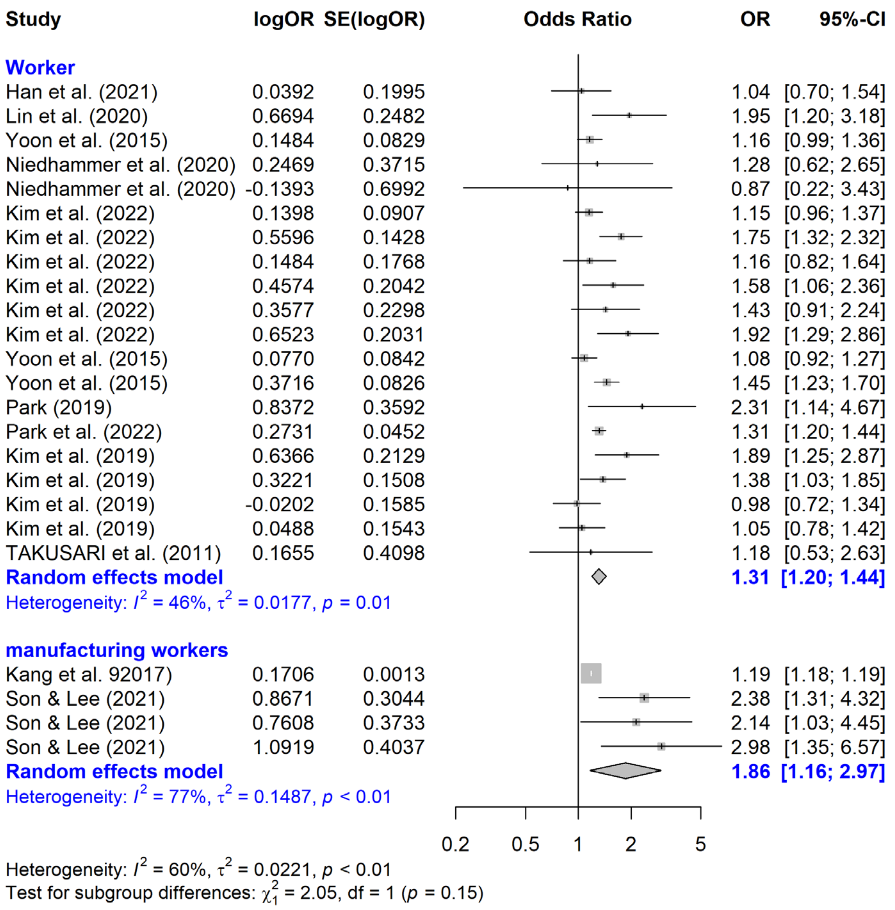

Supplementary Figure F7. Meta-analysis of differences in suicidal ideation by industry type

Supplementary table S 8. Non-adjusted OR between Shift work and suicide Ideation

	Non-adjusted OR (Day or regular vs Shift or Night)			
	OR	95% CI	N_{OR}	I^2(%)
Overall analysis	1.205	1.143 - 1.269	17	35.2
Sub-group				
Gender				
Men only	1.326	1.150 - 1.528	8	49.9
Women only	1.218	1.150 - 1.528	11	70.4
Working Type				
Shift Working	1.275	1.130 - 1.439	13	46.5
Fixed Evening	1.185	0.966 - 1.454	2	38.6
Fixed Night	1.218	1.065 - 1.393	2	0
Worker				
Manufacturing	1.601	1.112 - 2.306	3	0
General	1.197	1.135 - 1.262	14	34.7
Geographic region				
Asia	1.202	1.140 - 1.267	14	41.3
Non-Asia	1.439	0.921 - 2.249	3	0

Appendix G: Sensitivity analyses

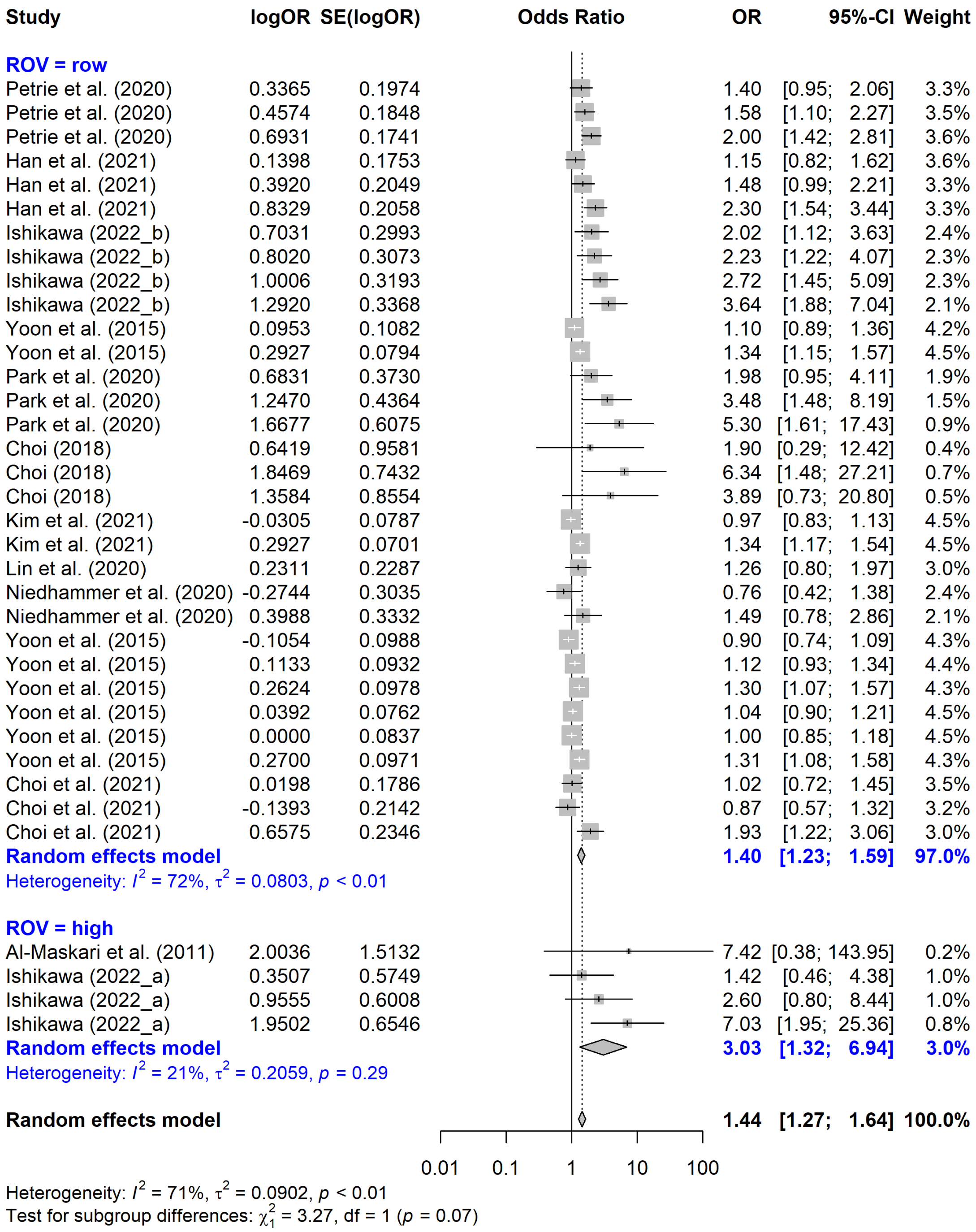

Supplementary Figure G1. Sensitivity analysis by Risk of bias on Long-working hours

Study	logOR	SE(logOR)	OR	95%-CI	Weight
**study_design = Cross sectional**					
Petrie et al. (2020)	0.2151	0.2624	1.24	[0.74; 2.07]	2.3%
Petrie et al. (2020)	0.3365	0.1974	1.40	[0.95; 2.06]	3.0%
Petrie et al. (2020)	0.4574	0.1848	1.58	[1.10; 2.27]	3.1%
Petrie et al. (2020)	0.6931	0.1741	2.00	[1.42; 2.81]	3.3%
Han et al. (2021)	0.1398	0.1753	1.15	[0.82; 1.62]	3.2%
Han et al. (2021)	0.3920	0.2049	1.48	[0.99; 2.21]	2.9%
Han et al. (2021)	0.8329	0.2058	2.30	[1.54; 3.44]	2.9%
Lin et al. (2020)	0.2311	0.2287	1.26	[0.80; 1.97]	2.6%
Ishikawa (2022_b)	0.7031	0.2993	2.02	[1.12; 3.63]	2.0%
Ishikawa (2022_b)	0.8020	0.3073	2.23	[1.22; 4.07]	1.9%
Ishikawa (2022_b)	1.0006	0.3193	2.72	[1.45; 5.09]	1.8%
Ishikawa (2022_b)	1.2920	0.3368	3.64	[1.88; 7.04]	1.7%
Yoon et al. (2015)	0.0953	0.1082	1.10	[0.89; 1.36]	4.1%
Yoon et al. (2015)	0.2927	0.0794	1.34	[1.15; 1.57]	4.4%
Niedhammer et al. (2020)	-0.2744	0.3035	0.76	[0.42; 1.38]	2.0%
Niedhammer et al. (2020)	0.3988	0.3332	1.49	[0.78; 2.86]	1.7%
Al-Maskari et al. (2011)	2.0036	1.5132	7.42	[0.38; 143.95]	0.1%
Park et al. (2020)	0.6831	0.3730	1.98	[0.95; 4.11]	1.5%
Park et al. (2020)	1.2470	0.4364	3.48	[1.48; 8.19]	1.2%
Ishikawa (2022_a)	0.3507	0.5749	1.42	[0.46; 4.38]	0.8%
Choi et al. (2021)	0.1398	0.1753	1.15	[0.82; 1.62]	3.2%
Choi et al. (2021)	0.3646	0.1856	1.44	[1.00; 2.07]	3.1%
Choi (2018)	0.6419	0.9581	1.90	[0.29; 12.42]	0.3%
Choi (2018)	1.8469	0.7432	6.34	[1.48; 27.21]	0.5%
Choi (2018)	1.3584	0.8554	3.89	[0.73; 20.80]	0.4%
Yoon et al. (2015)	-0.1054	0.0988	0.90	[0.74; 1.09]	4.2%
Yoon et al. (2015)	0.1133	0.0932	1.12	[0.93; 1.34]	4.3%
Yoon et al. (2015)	0.2624	0.0978	1.30	[1.07; 1.57]	4.2%
Yoon et al. (2015)	0.0392	0.0762	1.04	[0.90; 1.21]	4.4%
Yoon et al. (2015)	0.0000	0.0837	1.00	[0.85; 1.18]	4.4%
Yoon et al. (2015)	0.2700	0.0971	1.31	[1.08; 1.58]	4.2%
Kim et al. (2021)	0.0070	0.0015	1.01	[1.00; 1.01]	4.9%
Li et al. (2022)	0.3549	0.1840	1.43	[0.99; 2.05]	3.1%
Li et al. (2022)	0.6826	0.2190	1.98	[1.29; 3.04]	2.7%
**Random effects model**			**1.40**	**[1.25; 1.57]**	**90.6%**
Heterogeneity: $I^2 = 79\%$, $\tau^2 = 0.0632$, $p < 0.01$					
**study_design = prospective cohort**					
Tyssen et al. (2001)	-0.0619	0.0272	0.94	[0.89; 0.99]	4.8%
Malone et al. (2021)	0.2927	0.0624	1.34	[1.19; 1.51]	4.6%
**Random effects model**			**1.12**	**[0.79; 1.58]**	**9.4%**
Heterogeneity: $I^2 = 96\%$, $\tau^2 = 0.0605$, $p < 0.01$					
**Random effects model**			**1.37**	**[1.23; 1.52]**	**100.0%**

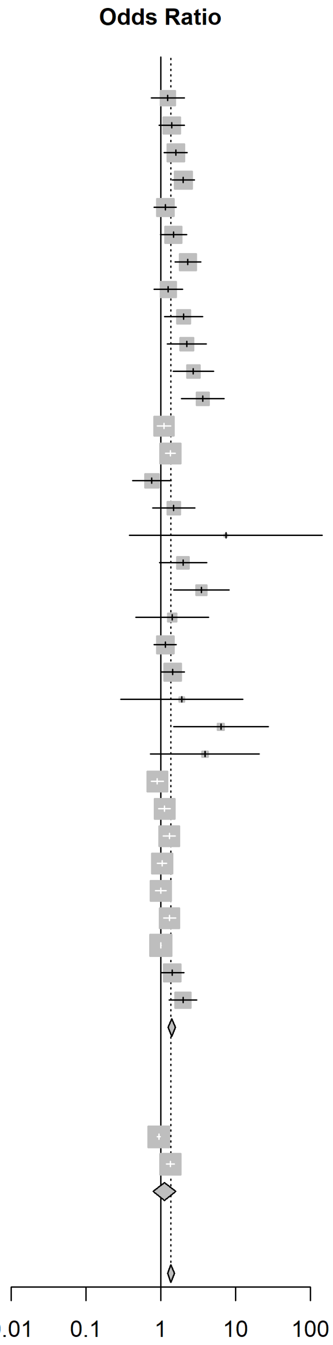

Heterogeneity: $I^2 = 81\%$, $\tau^2 = 0.0617$, $p < 0.01$
Test for subgroup differences: $\chi^2_1 = 1.46$, df = 1 ($p = 0.23$)

Supplementary Figure G2. Sensitivity analysis by Study design on Long-working hours

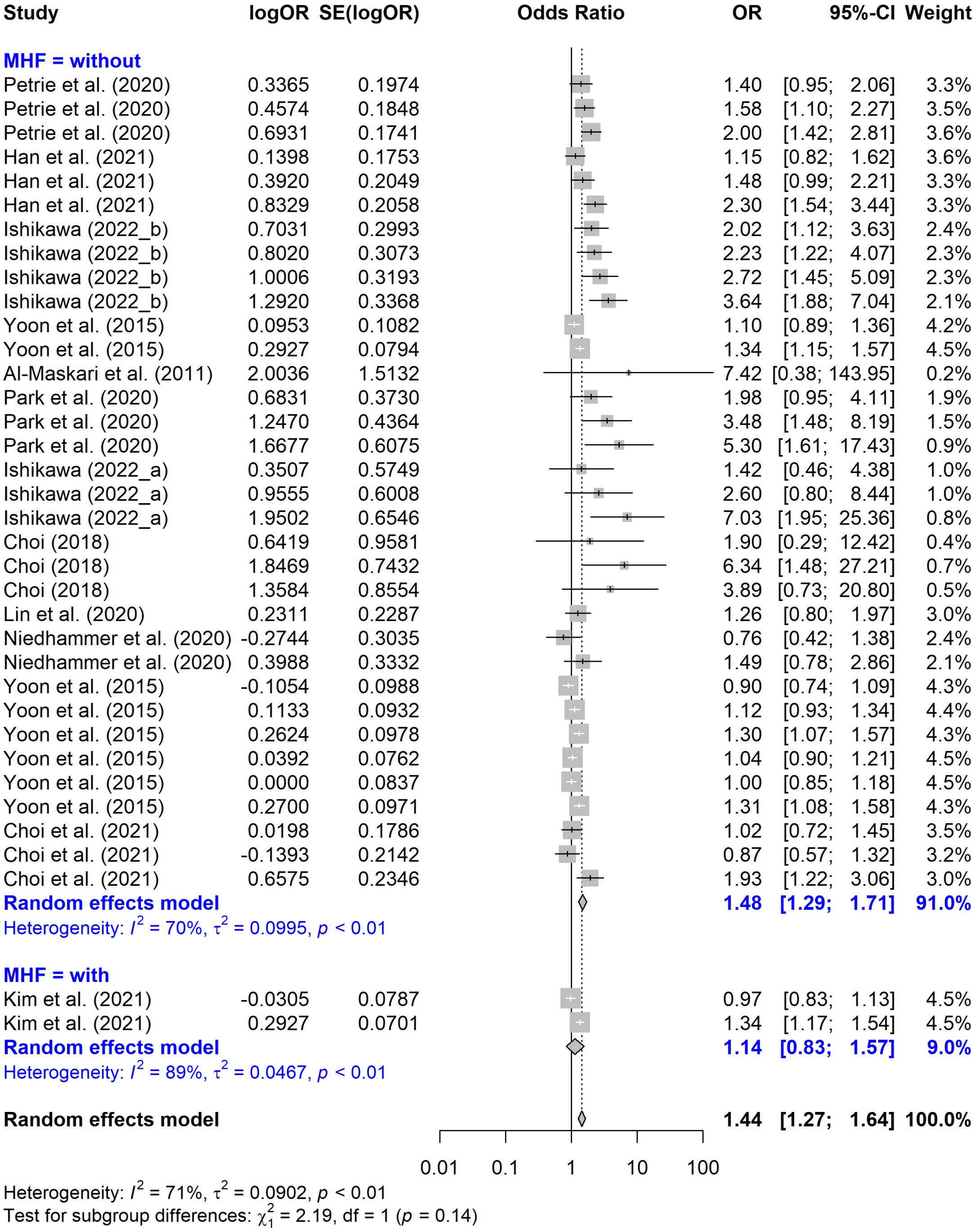

Supplementary Figure G3. Sensitivity analysis with and without adjustment for mental health factors on Long-working hours

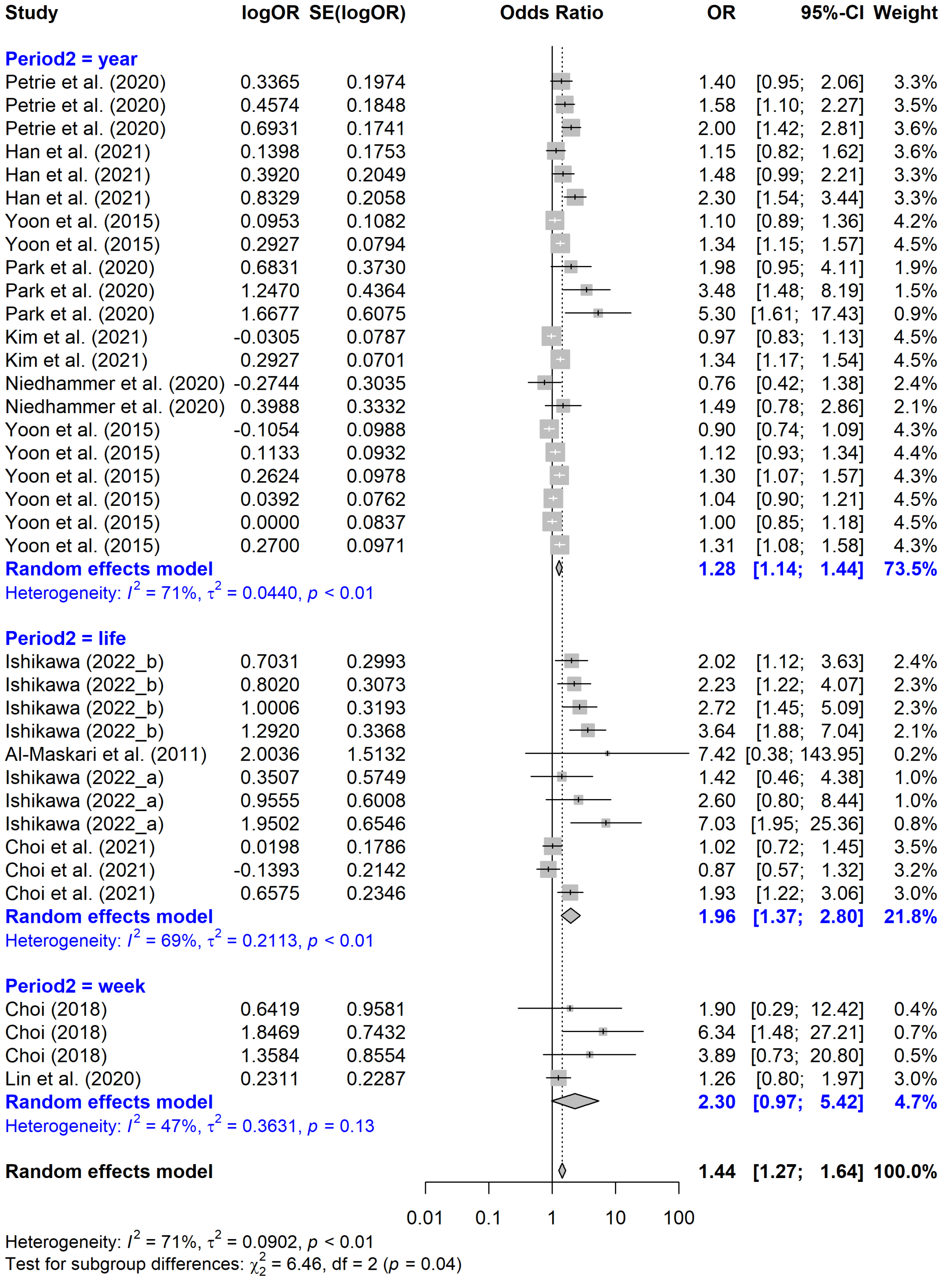

Supplementary Figure G4. Sensitivity analysis by Period for outcome index on Long-working hours

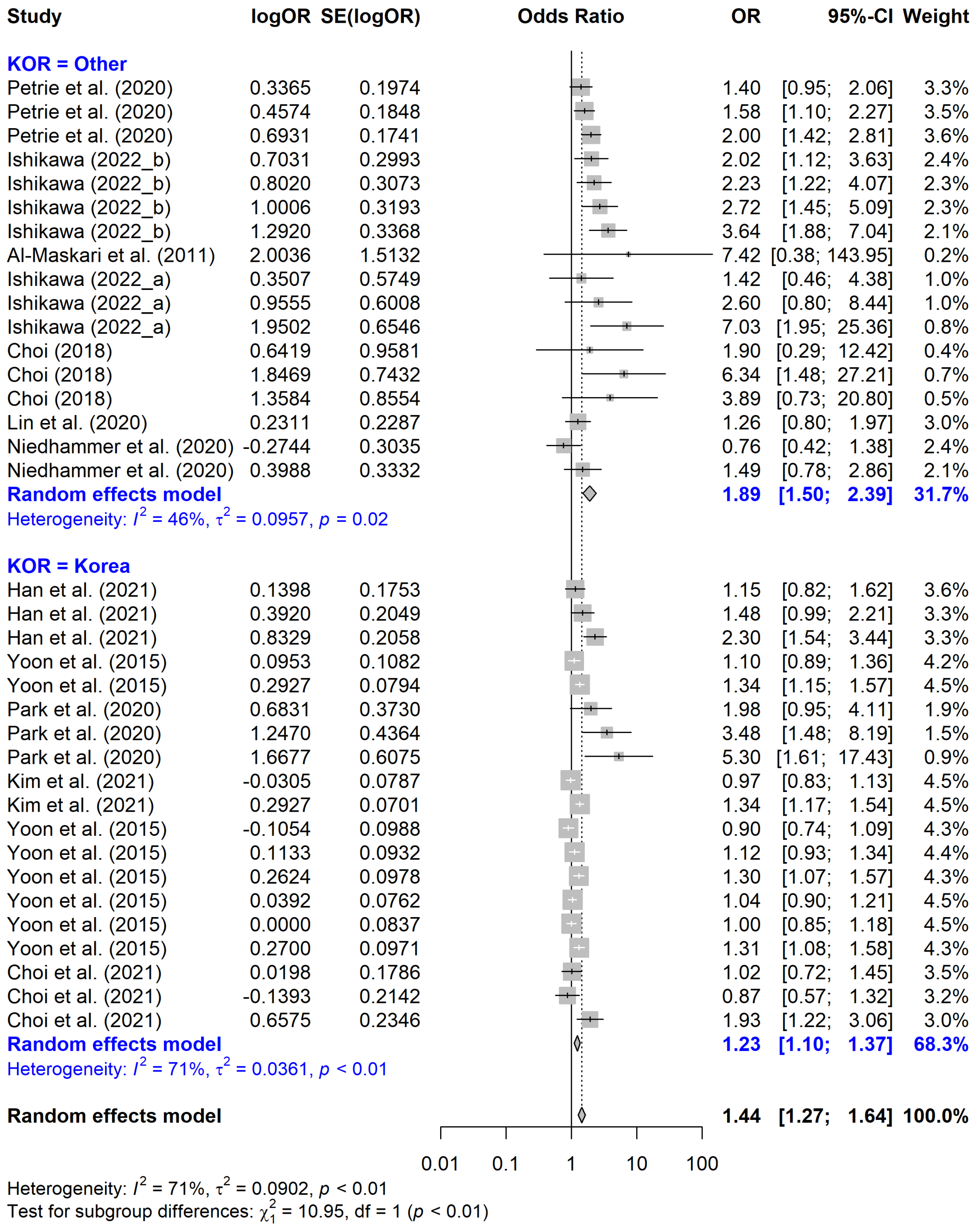

Supplementary Figure G5. Sensitivity analysis by Study conducted Country on Long-working hours

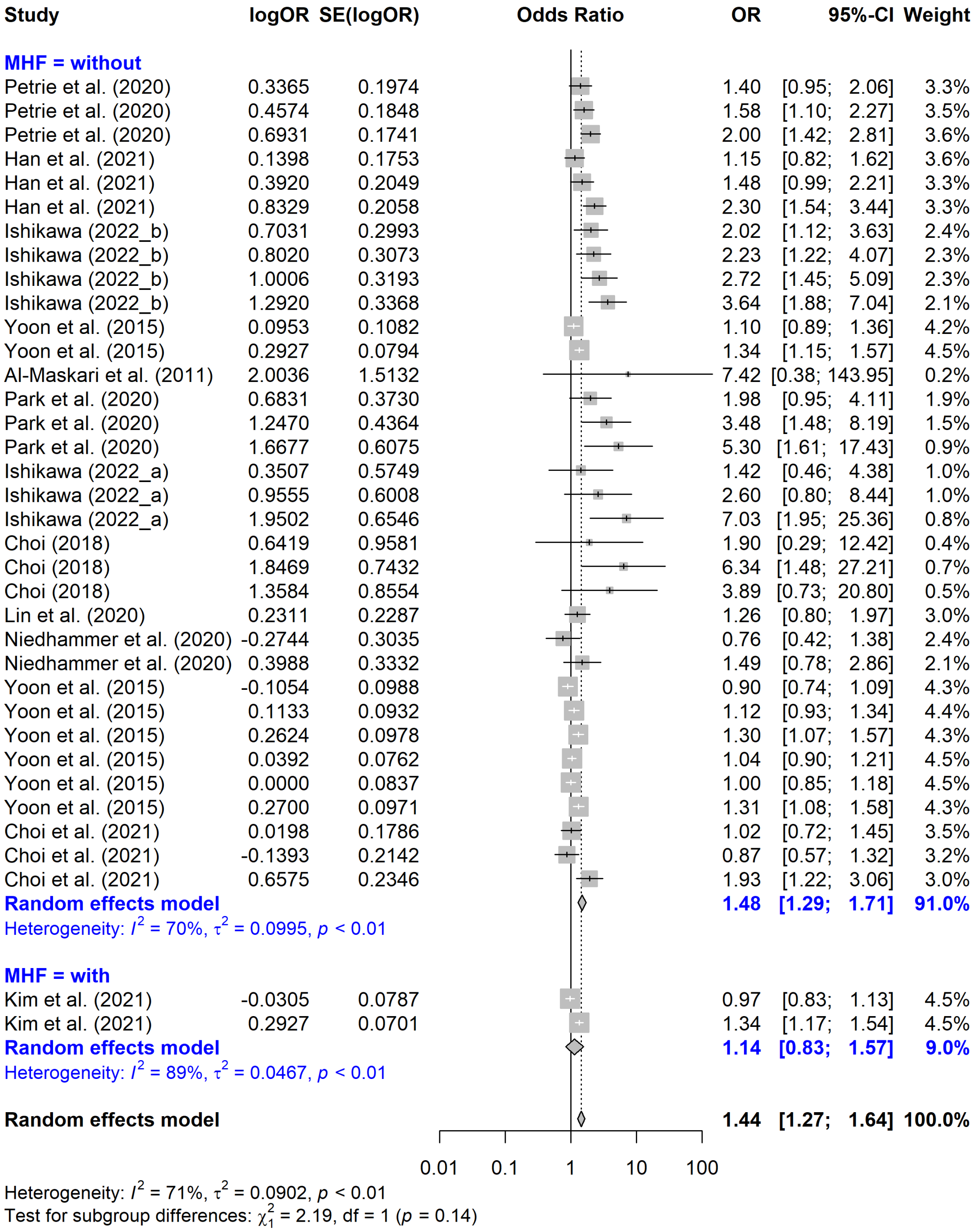

Supplementary Figure G6. Sensitivity analysis with and without adjustment for mental health factors on shift work

Study	logOR	SE(logOR)	OR	95%-CI	Weight
**Period2 = year**					
Han et al. (2021)	0.0392	0.1995	1.04	[0.70; 1.54]	3.5%
Kang et al. (2017)	0.1706	0.0013	1.19	[1.18; 1.19]	9.9%
Yoon et al. (2015)	0.1484	0.0829	1.16	[0.99; 1.36]	7.5%
Niedhammer et al. (2020)	0.2469	0.3715	1.28	[0.62; 2.65]	1.4%
Niedhammer et al. (2020)	-0.1393	0.6992	0.87	[0.22; 3.43]	0.4%
Kim et al. (2022)	0.1398	0.0907	1.15	[0.96; 1.37]	7.2%
Kim et al. (2022)	0.5596	0.1428	1.75	[1.32; 2.32]	5.1%
Kim et al. (2022)	0.1484	0.1768	1.16	[0.82; 1.64]	4.1%
Kim et al. (2022)	0.4574	0.2042	1.58	[1.06; 2.36]	3.4%
Kim et al. (2022)	0.3577	0.2298	1.43	[0.91; 2.24]	2.9%
Kim et al. (2022)	0.6523	0.2031	1.92	[1.29; 2.86]	3.4%
Yoon et al. (2015)	0.0770	0.0842	1.08	[0.92; 1.27]	7.5%
Yoon et al. (2015)	0.3716	0.0826	1.45	[1.23; 1.70]	7.5%
Son & Lee (2021)	0.8671	0.3044	2.38	[1.31; 4.32]	1.9%
Son & Lee (2021)	0.7608	0.3733	2.14	[1.03; 4.45]	1.3%
Son & Lee (2021)	1.0919	0.4037	2.98	[1.35; 6.57]	1.2%
Park (2019)	0.8372	0.3592	2.31	[1.14; 4.67]	1.4%
Park et al. (2022)	0.2731	0.0452	1.31	[1.20; 1.44]	9.0%
Kim et al. (2019)	0.6366	0.2129	1.89	[1.25; 2.87]	3.2%
Kim et al. (2019)	0.3221	0.1508	1.38	[1.03; 1.85]	4.9%
Kim et al. (2019)	-0.0202	0.1585	0.98	[0.72; 1.34]	4.6%
Kim et al. (2019)	0.0488	0.1543	1.05	[0.78; 1.42]	4.7%
**Random effects model**			**1.32**	**[1.21; 1.45]**	**96.2%**
Heterogeneity: $I^2 = 60\%$, $\tau^2 = 0.0199$, $p < 0.01$					
**Period2 = week**					
Lin et al. (2020)	0.6694	0.2482	1.95	[1.20; 3.18]	2.6%
**Period2 = life**					
TAKUSARI et al. (2011)	0.1655	0.4098	1.18	[0.53; 2.63]	1.1%
**Random effects model**			**1.34**	**[1.22; 1.47]**	**100.0%**

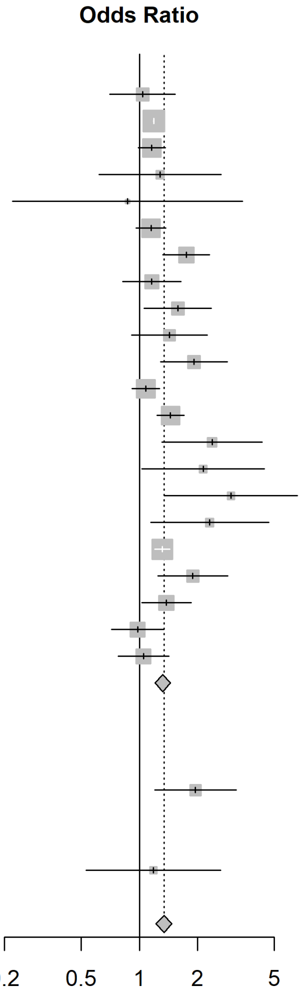

Heterogeneity: $I^2 = 60\%$, $\tau^2 = 0.0221$, $p < 0.01$
Test for subgroup differences: $\chi^2_2 = 2.46$, df = 2 ($p = 0.29$)

Supplementary Figure G7. Sensitivity analysis by Period for outcome index on shift work

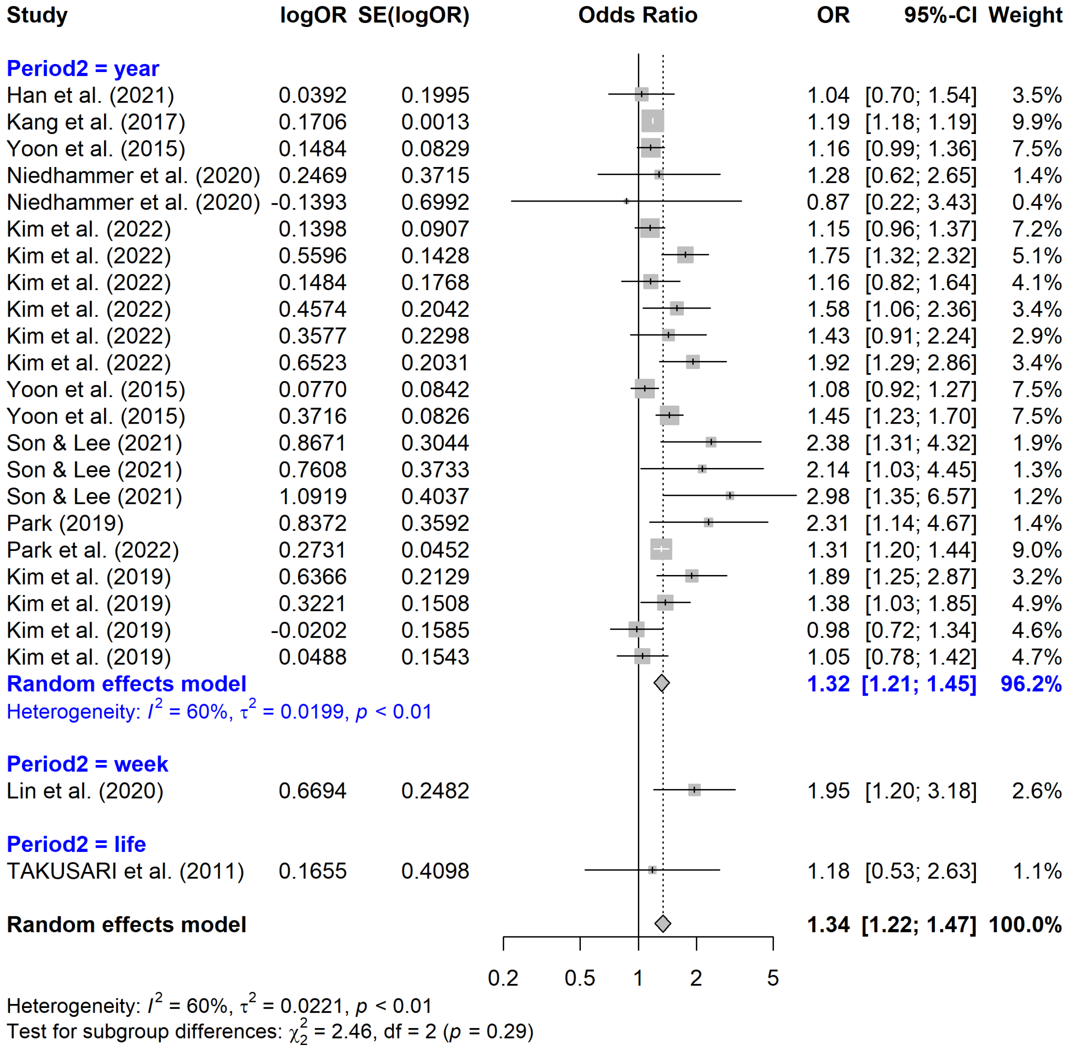

Supplementary Figure G8. Sensitivity analysis by Study conducted Country on shift work
